# Riluzole Attenuates L-DOPA-Induced Abnormal Involuntary Movements Through Decreasing CREB1 Activity: Insights from a Rat Model

**DOI:** 10.1007/s12035-018-1433-x

**Published:** 2018-11-27

**Authors:** Luca Pagliaroli, Joanna Widomska, Ester Nespoli, Tobias Hildebrandt, Csaba Barta, Jeffrey Glennon, Bastian Hengerer, Geert Poelmans

**Affiliations:** 1 Institute of Medical Chemistry, Molecular Biology and Pathobiochemistry, Semmelweis University, Budapest, Hungary; 2 Department of Cognitive Neuroscience, Donders Institute for Brain, Cognition and Behaviour, Radboud University Medical Center, Nijmegen, The Netherlands; 3 CNS Department, Boehringer Ingelheim Pharma GmbH & Co. KG, Biberach an der Riss, Germany; 4 Department of Child and Adolescent Psychiatry/Psychotherapy, University of Ulm, Ulm, Germany; 5 Target Discovery, Boehringer Ingelheim Pharma GmbH & Co. KG, Biberach an der Riss, Germany; 6 Department of Human Genetics, Radboud University Medical Center, PO Box 9101, 6500 HB Nijmegen, The Netherlands

**Keywords:** AIMs, Dyskinesia, 6-OHDA-lesioned rats, Riluzole, CREB1

## Abstract

Chronic administration of L-DOPA, the first-line treatment of dystonic symptoms in childhood or in Parkinson’s disease, often leads to the development of abnormal involuntary movements (AIMs), which represent an important clinical problem. Although it is known that Riluzole attenuates L-DOPA-induced AIMs, the molecular mechanisms underlying this effect are not understood. Therefore, we studied the behavior and performed RNA sequencing of the striatum in three groups of rats that all received a unilateral lesion with 6-hydroxydopamine in their medial forebrain bundle, followed by the administration of saline, L-DOPA, or L-DOPA combined with Riluzole. First, we provide evidence that Riluzole attenuates AIMs in this rat model. Subsequently, analysis of the transcriptomics data revealed that Riluzole is predicted to reduce the activity of CREB1, a transcription factor that regulates the expression of multiple proteins that interact in a molecular landscape involved in apoptosis. Although this mechanism underlying the beneficial effect of Riluzole on AIMs needs to be confirmed, it provides clues towards novel or existing compounds for the treatment of AIMs that modulate the activity of CREB1 and, hence, its downstream targets.

**Electronic supplementary material:**

The online version of this article (10.1007/s12035-018-1433-x) contains supplementary material, which is available to authorized users.

## Introduction

Chronic administration of the dopamine precursor L-DOPA—the first-line treatment of dystonic symptoms manifesting in childhood or in Parkinson’s disease (PD) [1]—often leads to the development of so-called abnormal involuntary movements (AIMs). In humans, L-DOPA-induced AIMs are referred to as L-DOPA-induced dyskinesia (LID) [2], which represents an important clinical problem as approximately 90% of PD patients develop LID within 10 years of starting L-DOPA treatment. As a result, the prescription of L-DOPA to PD patients is often delayed to minimize the risk of developing LID [3].

The pathogenic mechanisms underlying AIMs and clinical LID are still largely unclear. Literature evidence indicates that chronic L-DOPA administration affects multiple neurotransmitter systems [4–6], but mainly the dopaminergic system, i.e., L-DOPA activates striatal dopamine 1 receptors (D1Rs), resulting in the overstimulation of the “direct” nigrostriatal pathway [7, 8]. In turn, this hypersensitivity of D1Rs in striatal neurons triggers a number of downstream signaling cascades involving—among others—cyclic AMP (cAMP)-activated protein kinase A (PKA) [9, 10], the phosphatase inhibitor DARPP-32 [10, 11], and the ERK1 and ERK2 (ERK1/2) kinases [12, 13] that are subsequently implicated in the development of LID through modulating gene expression and function [14, 15]. In this respect, previous transcriptomic and methylation studies in rodent models indeed indicate that AIMs/LID are associated with aberrant gene expression through transcription factors, such as CREB1, proteins from the AP-1 complex [9, 16] (such as FOSB) [17], and GADD45 family proteins [17].

Recently, Riluzole has been suggested as a candidate drug to treat AIMs/LID. Indeed, several studies on rodent models have provided evidence for the efficacy of Riluzole in the management of AIMs [2, 18, 19]. As for human studies, Riluzole showed anti-dyskinetic effects without affecting the anti-parkinsonian action of L-DOPA in a small pilot study [20], but it was ineffective in another clinical trial [21]. Riluzole essentially has anti-glutamatergic properties—including effects on glutamate release, uptake, and receptor signaling [22–25], and it protects against glutamate-induced activation of kinases, such as ERK1/2 [26]. More specifically, although Riluzole has properties resembling those of a competitive NMDA receptor antagonist, its effects on reducing glutamatergic neurotransmission are more indirect [27]. Indeed, the anti-glutamatergic effects of Riluzole could result from a reduction of glutamate release from synapses through an inhibition of voltage-gated sodium channels [28], a reduction in the post-synaptic calcium influx mediated by P/Q-type calcium channels [29], a reduction in glutamine import into synapses (with glutamine being a precursor for the releasable glutamate pool) [30], or through interfering with the size of the readily releasable glutamate pool [31]. Further, animal model studies suggest that Riluzole is not selective for glutamate but also reduces the release of acetylcholine, dopamine, and, to a lesser extent, serotonin through mechanisms independent of glutamate receptors [32]. However, a detailed understanding of the molecular mechanism(s) underlying the protective effect of Riluzole on AIMs/LID is essentially lacking.

In this study, we aimed to elucidate the molecular mechanisms underlying the beneficial effect of Riluzole on AIMs. Therefore, we studied the behavior and performed RNA sequencing of the striatum, followed by gene expression analysis and comparison in three groups of rats. All rats received a unilateral lesion with 6-hydroxydopamine (6-OHDA) in their medial forebrain bundle (MFB)—resulting in striatal dopaminergic hypersensitivity—after which they were administered saline, L-DOPA (which constitutes a rat model of AIMs), or L-DOPA combined with Riluzole. We found that Riluzole attenuates L-DOPA-induced AIMs in the rat model. In addition, further analysis indicated that Riluzole is predicted to reduce the activity of the transcription factor CREB1, the key hub in a landscape of interacting proteins that are involved in regulating neuronal apoptosis.

## Methods

### Animals and Ethical Approvals

Juvenile male Wistar rats (RjHan:WI) were used in this study. Rats were obtained from our provider (Janvier, Le Genest-St Isle, France) at the age of 14 days together with their mothers. In total, 5 juvenile rats and a mother were housed together with free access to food and water, under a 12-h light and dark cycle in temperature- and humidity-controlled rooms. All animals were habituated to their housing conditions for one week. Animal housing and experiments were approved by the appropriate institutional governmental agency (Regierungspräsidium Tübingen, Germany) and performed in an AAALAC (Association for Assessment and Accreditation of Laboratory Animal Care International)-accredited facility, following the European Convention for Animal Care and Use of Laboratory Animals.

### Stereotaxic Surgery

After 1 week of habituation at post-natal day (PND) 21 (*n* = 48), the rats were separated from their mothers and underwent stereotaxic surgery with 6-hydroxydopamine hydrobromide (6-OHDA). Further details are provided in the Supplementary methods.

### Animal Treatment

Two weeks after stereotaxic surgery, the rats were randomly allocated to 4 groups (*n* = 12 animals per group) associated to different pharmacological treatments, at the same conditions: the first group was chronically administered (6 times in 2 weeks, intra-peritoneally (i.p.)) with a solution of L-DOPA methyl ester hydrochloride and benserazide (Sigma-Aldrich Chemie GmbH, Germany) in saline (6/15 mg/kg, i.p.). The second group (control) was administered with saline. The third group was chronically administered (6 times in 2 weeks) with Riluzole (Sigma-Aldrich Chemie GmbH, Germany) dissolved in 1% Tween (6 mg/kg, i.p.) and L-DOPA/benserazide (6/15 mg/kg, i.p.). The fourth (6-OHDA-lesioned) group followed the same procedure as group 1 and was used to verify the presence of the lesion by quantification of dopamine (DA) levels in striatal tissue using high-performance liquid chromatography (HPLC) coupled to electrochemical detection (ECD).

### Phenotype Scoring

Four weeks after surgery (PND 49), the rats were administered their treatment and were individually observed for 1 min every 20 min. Abnormal involuntary movements (AIMs) of the limb, mouth, and body axis were assessed according to the widely used AIMs rating scale [33]. Briefly, each type of movement was scored on a scale from 0 to 4 (0 = absent; 1 = occasional; 2 = frequent; 3 = continuous but interrupted by sensory distraction; 4 = continuous, severe, not interrupted by sensory distraction). The sum of the independent scores of each body part per time point gave the total AIMs score.

### Animal Sacrifice and Sample Extraction

**Fig. 1:**
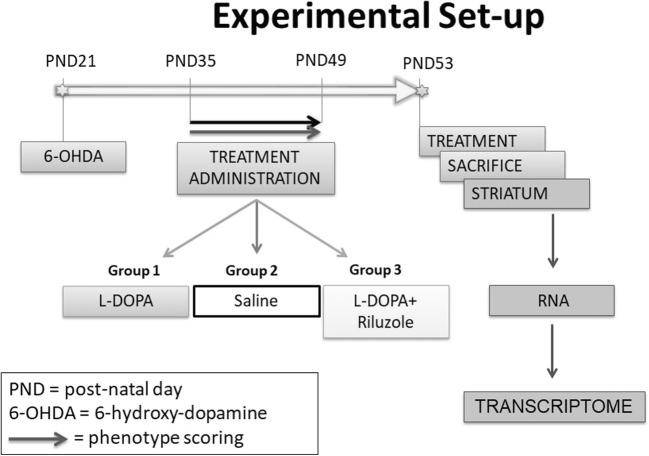
Schematic diagram of the protocol employed including the time course of the experiment and the different assays that were used

At PND 53, rats were sacrificed 2 h after the administration of a final treatment. Rats were anesthetized for 60 s in 5% isoflurane and quickly decapitated by guillotine. Following brain removal, lesioned and unlesioned striata were extracted, snapped-frozen in liquid nitrogen, and stored at − 80 °C. A schema of the experimental procedure is outlined in Fig. 1.

### Striatal DA Determination Through HPLC

DA levels in 12 6-OHDA-lesioned striata compared to their contralateral controls were quantified using HPLC coupled to ECD. Further details are provided in the Supplementary methods.

### Behavioral Testing: Statistical Analysis

Time-course data were analyzed for statistical significance using two-way analysis of variance (ANOVA) followed by Tukey’s multiple comparison test. Dopaminergic HPLC quantification in lesioned versus unlesioned striata was analyzed using a Wilcoxon matched-pairs signed-ranked test.

### RNA Sequencing and Data Analysis

Total RNA was extracted from striatum tissue from 8 animals from each group using the QiagenAllPrep Kit (Qiagen, Hamburg, Germany), according to the manufacturer’s instructions. RNA was further quantified using the NanoDrop 1000 Spectrophotometer and Bioanalyzer 2100 (Agilent). Samples with RIN value above 8.0 were used for transcriptome analysis. The sequencing library preparation was done using 200 ng of total RNA input with the TrueSeq RNA Sample Prep Kit v3-Set B (RS-122-2002, Illumina Inc., San Diego, CA) producing a 275 bp fragment including adapters in average size. In the final step before sequencing, 12 individual libraries were normalized and pooled together using the adapter indices supplied by the manufacturer. Pooled libraries were clustered on the cBot Instrument from Illumina using the TruSeq SR Cluster Kit v3—cBot—HS (GD-401-3001, Illumina Inc., San Diego, CA), and sequencing was then performed as 78 bp, single reads and 7 bases index read on an Illumina HiSeq3000 instrument using the TruSeq SBS Kit HS—v3 (50-cycle) (FC-401-3002, Illumina Inc., San Diego, CA). Reads were aligned to the rat genome using the STAR Aligner v2.5.2a [34] with corresponding Ensembl 84 reference genome (http://www.ensembl.org). Sequenced read quality was checked with FastQC v0.11.2 (http://www.bioinformatics.babraham.ac.uk/projects/fastqc/), and alignment quality metrics were calculated using RNASeQC v1.18 [35]. Following read alignment, duplication rates of the RNA sequencing samples were computed with bamUtil v1.0.11 to mark duplicate reads and the dupRadar v1.4 Bioconductor R package for assessment [36]. Gene expression profiles were quantified using Cufflinks software version 2.2.1 [37] and the feature counts software package [38]. Transcripts with at least 10 reads in 8 samples were retained and further used to identify differentially expressed genes between the following three experimental groups: saline, L-DOPA, and L-DOPA + Riluzole. Statistical analyses were performed using R (www.r-project.org) and the Bioconductor package ([39], www.bioconductor.org) limma-voom [40]. The Benjamini-Hochberg’s method was used to correct for multiple testing, and only protein-coding genes with adjusted *p* value < 0.01, independent of magnitude of change, were considered as differentially expressed and used in the subsequent analyses. The Venn diagram of the final sets of significantly differentially expressed genes for the two comparisons was built with Venny [41]. To assess the effect of Riluzole on L-DOPA-regulated genes, we also looked at the overlap between the genes regulated by L-DOPA (L-DOPA vs. saline, comparison 1) and the genes regulated by Riluzole in the L-DOPA model (L-DOPA + Riluzole vs. L-DOPA, comparison 2). To quantify this overlap, we used the hypergeometric distribution test [42]:

$$ p\left(x|\ n,M,N\right)=\frac{\left(\genfrac{}{}{0pt}{}{M}{x}\right)\left(\genfrac{}{}{0pt}{}{N-M}{n-x}\right)}{\left(\genfrac{}{}{0pt}{}{N}{n}\right)} $$

This test calculates the chance of observing exactly *x* overlapping genes from a total of *n* differentially expressed genes by Riluzole in the L-DOPA model, with a total of *M* genes that were differentially expressed by L-DOPA and a total of *N* genes detected with RNAseq. The total number of unique genes detected with RNAseq (*N*) consists of genes detected in both comparisons, irrespective of their FC or expression *p* value. For all comparisons, only protein-coding genes were considered. To determine the correlation between the fold changes for the overlapping genes in the two comparisons, we created a scatter plot and calculated the Spearman’s correlation coefficient using the ggscatter function in R package ggpubr.

### Upstream Regulator and Gene Enrichment Analyses

Using Ingenuity Pathway Analysis (IPA; Qiagen Inc.), we performed upstream regulator and gene enrichment analyses. Based on the differentially expressed genes, IPA generates a list of “upstream regulators,” i.e., proteins or compounds that are able to explain observed gene expression changes in the input dataset. For each upstream regulator, IPA calculates a *p* value of overlap (measuring the statistical significance of the overlap between the dataset genes and all genes that are regulated by the upstream regulator) and a *z* score (reflecting the inhibition or activation of the upstream regulator-dependent effects on target gene expression). We selected the major upstream regulator with the best *z* score and *p* value of overlap for the two comparisons—i.e., L-DOPA vs. saline and L-DOPA + Riluzole vs. L-DOPA—and used the target genes of this upstream regulator for further analyses. In the gene enrichment analysis, IPA assigns genes and their corresponding mRNAs/proteins to functional (sub)-categories, i.e., “canonical pathways” and “biofunctions,” with the latter including “diseases and disorders” and “molecular and cellular functions.”

### Molecular Landscape Building

Following the upstream regulator and enrichment analyses, we searched the literature for the functions and interactions of all proteins encoded by the target genes of the major upstream regulator and expressed in the opposite direction in the two comparisons, i.e., L-DOPA vs. saline and L-DOPA + Riluzole vs. L-DOPA. First, we used the UniProt Knowledge Base ([43], http://www.uniprot.org) to gather basic information on the functions of all target genes and their encoded proteins. Subsequently and starting with the interactions reported by IPA, we used PubMed (http://www.ncbi.nlm.nih.gov/pubmed) to search for all functional, experimental evidence-based interactions between the proteins. Based on all gathered information, we generated a protein interaction landscape. The figure depicting this landscape was made using the drawing program Serif DrawPlus version 4.0 (www.serif.com).

### qPCR Validation of Selected CREB1 Targets

To provide an additional validation of the RNA sequencing data, we performed qPCR to assess and compare the mRNA expression levels of 10 randomly chosen CREB1 targets in each of the samples of the groups investigated in this study (saline, L-DOPA, L-DOPA + Riluzole). RNA from the same samples used for the RNA sequencing was reverse-transcribed to cDNA using the RT^2^ First-Strand Kit (Qiagen, Cat. number 330404) according to the manufacturer’s instructions. Three-step qPCR (95 °C for 10 min, followed by 40 two-step cycles at 95 °C for 15 s and 60 °C for 30 s, and the generation of melting curves from 70 to 95 °C; Rotor-Gene Q 1000, Qiagen) was performed using RT^2^ SYBR Green ROX™ qPCR Mastermix (Qiagen, Cat. number 330521) and RT^2^ qPCR primer Assays provided by Qiagen. The housekeeping genes *Bcap29* and *Cdkn1b* were used as reference for normalization of gene expression, and a Student’s *t* test was used to assess statistical significance. The full list of primers is specified in Supplementary Table 1.

## Results

### Assessment of Striatal DA Depletion by Assaying Its Content

To confirm the degree of dopaminergic denervation in the unilateral 6-OHDA-lesioned rats, total (intracellular and extracellular) dopamine (DA) tissue content in the ipsilateral (lesioned) and contralateral (control) striatum relative to the lesion was assayed by HPLC coupled to ECD. 6-OHDA lesion induced a marked decrease (76%) in DA concentration in the ipsilateral striatum compared with the contralateral side (Supplementary Fig. 1).

### Behavioral Effects: Induction of AIMs by L-DOPA in 6-OHDA-Lesioned Rats and Their Mitigation by Riluzole

**Fig. 2:**
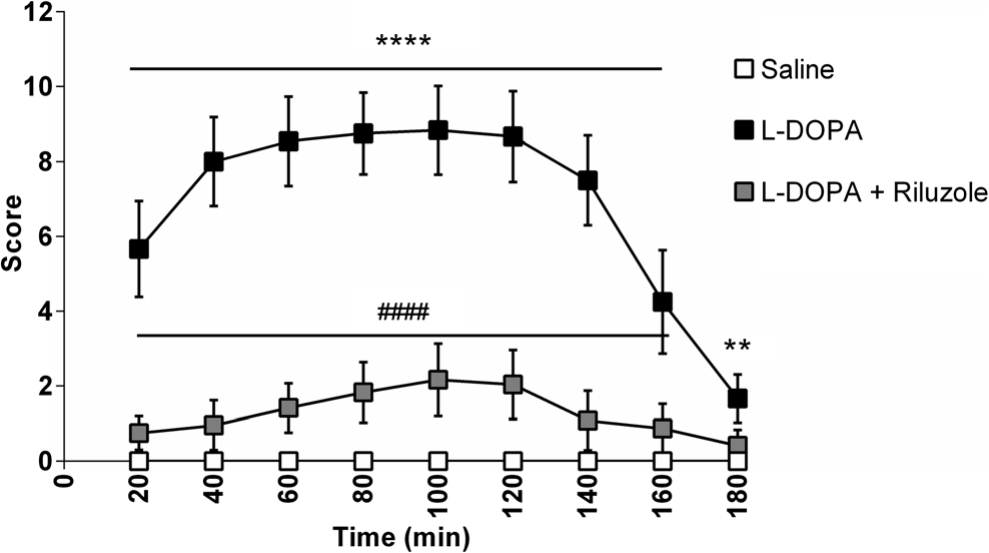
Induction of AIMs by L-DOPA in 6-OHDA-lesioned rats and their mitigation by Riluzole. Data shown as mean ± S.E.M. AIMs scores. Significant differences between the saline and L-DOPA-treated groups are indicated as **p < 0.01 and ****p < 0.001 and between the L-DOPA and L-DOPA + Riluzole-treated groups as ^####^*p* < 0.001

Intra-MFB 6-OHDA lesion plus 2 weeks of chronic L-DOPA exposure induced strong abnormal involuntary movements (*p* < 0.001), while 6-OHDA-lesioned rats given saline showed no abnormal motor phenotype. Chronic treatment with Riluzole together with L-DOPA was associated with fewer AIMs than L-DOPA alone (*p* < 0.001), as shown in Fig. 2**.**

### RNA Sequencing Data Analysis and Upstream Regulator/Gene Enrichment Analyses

Transcriptomic analysis was performed on the mRNA from the RNA samples isolated from the (lesioned) striatum of saline-, L-DOPA-, and L-DOPA + Riluzole-treated rats 2 h after the administration of a final injection. This allowed examination of the striatal mRNA gene expression profiles (and assessment of the counteracting effect of Riluzole) at the peak of L-DOPA effectiveness. The statistically significant differences in gene expression levels in striatum of L-DOPA- vs. saline-treated rats (comparison 1) and in L-DOPA + Riluzole- vs. L-DOPA-treated rats (comparison 2) are shown in Supplementary Data file 1.

Among the differentially expressed genes, 667 were differentially expressed only in comparison 1 and 1200 genes were differentially expressed only in comparison 2. Further, 465 genes were overlapping in that they were differentially expressed in both comparisons (Supplementary Fig. 2), and *all* of these genes were differentially expressed in the opposite direction in the two comparisons (i.e., genes that were upregulated by chronic L-DOPA treatment were downregulated by Riluzole co-administration and vice versa). The significance of the overlap in differentially expressed genes was calculated by using the hypergeometric distribution test, which showed that the overlap is much greater than would be expected based on random gene selection (*p* value = 1.01E−142).

As indicated in Supplementary Table 2, cAMP-responsive element binding protein 1 (CREB1) is the top upstream regulator of both the genes that were differentially expressed in comparison 1 and comparison 2, and in these comparisons, CREB1 is predicted to be activated and inhibited, respectively. In addition, CREB1 is the top upstream regulator of the 465 overlapping differentially expressed genes (see previous texts) and there is a highly significant, negative correlation (*r* = − 0.91, *p* value = 2.2E−16) between the fold changes for these genes—including the 58 direct CREB1 target genes—in the two comparisons (Supplementary Fig. 3).

Furthermore, gene enrichment analysis (Supplementary Table 3) revealed that the three sets of differentially expressed genes are significantly enriched for a number of “diseases and disorders” categories, including “epileptic seizure” (enriched in all three gene sets), as well as “disorder of basal ganglia” and “neuromuscular disease” (enriched in the genes from comparison 1). In addition, the gene sets are enriched for multiple “molecular and cellular functions” categories, such as “cell death/apoptosis” and “expression/transcription of RNA” (enriched in all three gene sets) as well as “development of neurons” (enriched in the genes from comparison 2).

### Molecular Landscape of CREB1 Targets

Subsequently, we further investigated the CREB1-mediated molecular mechanisms implicated in the counteractive effect of Riluzole on L-DOPA-induced changes in gene expression. Therefore, the set of 58 CREB1 target genes that were regulated in the opposite direction by L-DOPA and Riluzole (Table 1) was studied in more detail, and enrichment analysis of these 58 overlapping genes showed that “epileptic seizure” and “apoptosis” are the most significantly enriched “diseases and disorders” and “molecular and cellular functions” categories, respectively (Supplementary Table 4).

**Table 1:** CREB1 target genes that were significantly differentially expressed in comparisons L-DOPA vs. saline and L-DOPA + Riluzole vs. L-DOPA with the corresponding fold changes (FDR-corrected *p* value < 0.01). The genes in bold encode proteins that are included in our molecular landscape (Fig. 3)

CREB1 target gene	Description	L-DOPA vs. saline	L-DOPA + Riluzole vs. L-DOPA
***Arc***	Activity regulated cytoskeleton-associated protein	6.41	− 2.99
***Atf3***	Activating transcription factor 3	10.98	− 6.06
***Bag3***	BCL2-associated athanogene 3	1.80	− 1.73
***Ccnd3***	Cyclin D3	1.22	− 1.31
***Cdc37***	Cell division cycle 37	1.27	− 1.28
*Cdk19*	Cyclin-dependent kinase 19	− 1.22	1.18
***Cdkn1a***	Cyclin-dependent kinase inhibitor 1A	4.09	− 2.57
***Crem***	cAMP-responsive element modulator	1.73	− 1.52
***Crh***	Corticotropin-releasing hormone	6.32	− 4.62
*Csrnp1*	Cysteine- and serine-rich nuclear protein 1	3.06	− 1.88
***Dusp14***	Dual specificity phosphatase 14	4.15	− 2.44
***Egr4***	Early growth response 4	10.01	− 4.16
*Ehd4*	EH domain containing 4	1.30	− 1.30
***Fgf13***	Fibroblast growth factor 13	− 1.29	1.26
***Fos***	Fos proto-oncogene, AP-1 transcription factor subunit	12.99	− 4.94
***Fosb***	FosB proto-oncogene, AP-1 transcription factor subunit	17.17	− 4.96
***Frmd6***	FERM domain containing 6	2.16	− 1.59
*Gaa*	Glucosidase alpha, acid	1.46	− 1.34
***Gadd45b***	Growth arrest and DNA damage-inducible beta	3.43	− 1.76
***Gadd45g***	Growth arrest and DNA damage-inducible gamma	4.59	− 2.92
*Gpr3*	G protein-coupled receptor 3	8.19	− 3.95
***Hspa5***	Heat-shock protein family A (Hsp70) member 5	1.64	− 1.41
***Id1***	Inhibitor of DNA binding 1, HLH protein	1.44	− 1.57
*Igsf9b*	Immunoglobulin superfamily member 9B	1.93	− 1.63
***Inhba***	Inhibin beta A subunit	5.76	− 3.41
***Irs2***	Insulin receptor substrate 2	6.01	− 3.00
***Jun***	Jun proto-oncogene, AP-1 transcription factor subunit	2.45	− 1.70
***Junb***	JunB proto-oncogene, AP-1 transcription factor subunit	7.83	− 3.28
***Klf4***	Kruppel like factor 4	2.00	− 1.60
*Lmo1*	LIM domain only 1	1.25	− 1.23
***Midn***	Midnolin	2.52	− 2.34
*Ndufv1*	NADH:ubiquinone oxidoreductase core subunit V1	1.19	− 1.21
***Nfil3***	Nuclear factor, interleukin 3 regulated	2.08	− 1.51
***Npas4***	Neuronal PAS domain protein 4	5.50	− 5.07
***Nptx2***	Neuronal pentraxin 2	4.96	− 2.70
***Npy***	Neuropeptide Y	1.72	− 2.23
***Nr4a1***	Nuclear receptor subfamily 4 group A member 1	4.27	− 2.15
***Nr4a3***	Nuclear receptor subfamily 4 group A member 3	10.26	− 4.16
*Nrgn*	Neurogranin	1.56	− 1.36
*Pdxk*	Pyridoxal kinase	1.93	− 1.69
***Pdyn***	Prodynorphin	2.50	− 1.75
***Per1***	Period circadian clock 1	2.18	− 1.87
*Pim3*	Pim-3 proto-oncogene, serine/threonine kinase	1.41	− 1.39
***Plat***	Plasminogen activator, tissue type	1.47	− 1.48
***Ptgs2***	Prostaglandin-endoperoxide synthase 2	5.15	− 3.08
*Pvr*	Poliovirus receptor	2.84	− 2.34
*Rem2*	RRAD- and GEM-like GTPase 2	5.42	− 2.82
***Rheb***	Ras homolog enriched in brain	1.49	− 1.18
***Scg2***	Secretogranin II	2.90	− 1.69
*Sema7a*	Semaphorin 7A (John Milton Hagen blood group)	1.30	− 1.27
***Sertad1***	SERTA domain containing 1	2.83	− 1.74
***Sh3kbp1***	SH3 domain containing kinase binding protein 1	− 1.15	1.13
***Sik1***	Salt-inducible kinase 1	3.10	− 2.08
*Slc32a1*	Solute carrier family 32 member 1	1.79	− 1.52
***Srxn1***	Sulfiredoxin 1	6.08	− 3.28
***Stat3***	Signal transducer and activator of transcription 3	1.13	− 1.13
***Tac1***	Tachykinin precursor 1	2.82	− 1.92
***Vegfa***	Vascular endothelial growth factor A	1.33	− 1.30

**Fig. 3:**
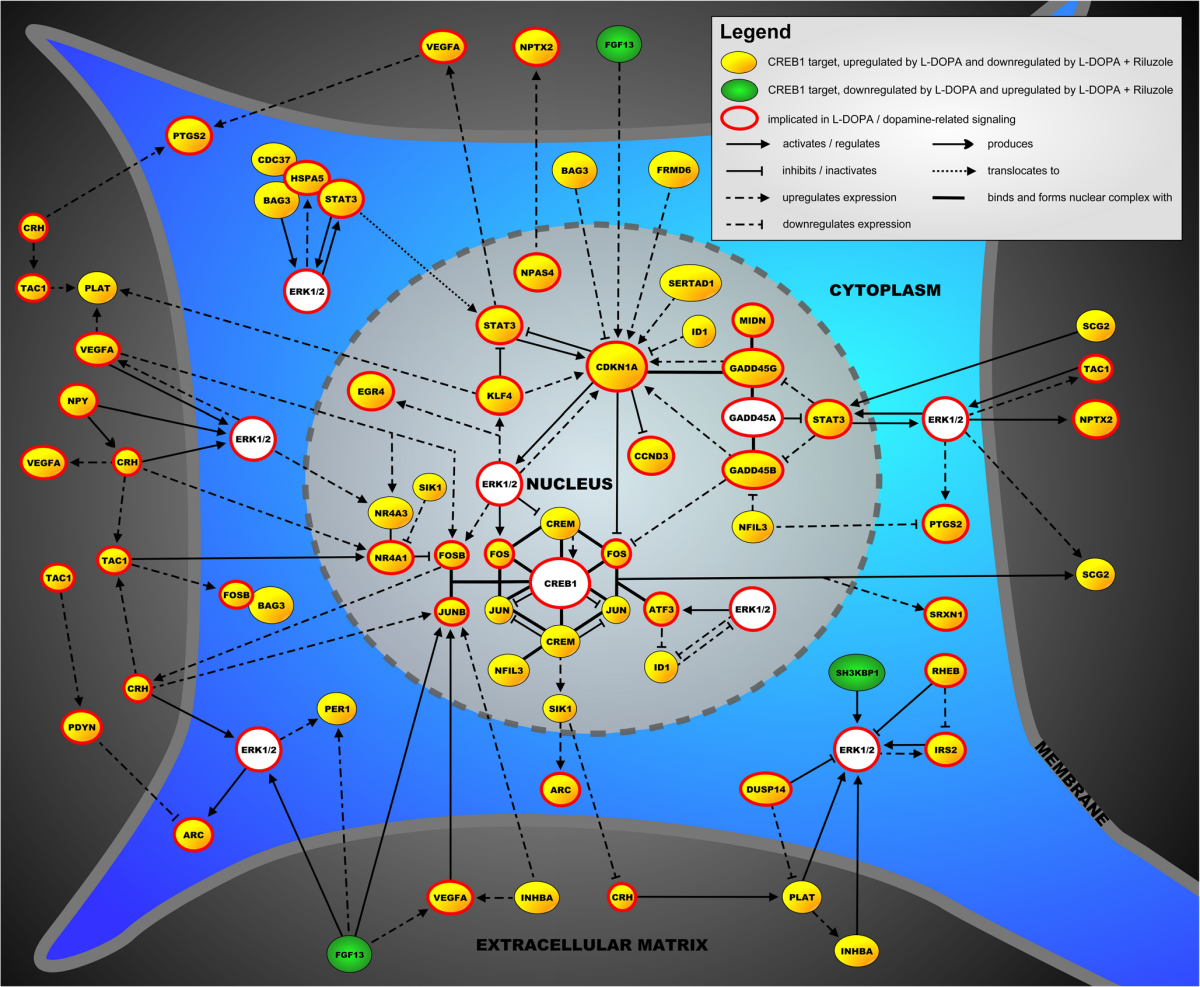
The molecular landscape is located in a neuron and shows the functional interactions between proteins encoded by 43 of the 58 CREB1 target genes regulated in the opposite direction by L-DOPA and after Riluzole co-administration in our AIMs model

The proteins encoded by 43 of the 58 CREB1 target genes could be placed into a molecular landscape (Fig. 3). In the supplementary materials (Supplementary materials), all the protein interactions in this landscape are described in great detail. That being said, we here give a succinct description of the main processes and signaling cascades in the landscape. Importantly, as the main signaling “hub” in the landscape, CREB1 is a transcription factor that regulates the expression of many target genes involved in neuronal processes, such as neuronal survival, differentiation, and development. Further, there are three main landscape cascades in the nucleus. First, the AP-1 transcription factor complex—consisting of JUN, FOS, JUNB, and FOSB—binds and interacts with CREB1 and is regulated by and regulates the expression of a number of other landscape proteins, e.g., ATF3 and CREM. In addition, a complex of two functionally related transcription factors—NR4A1 and NR4A3, which regulate neuronal survival downstream of CREB1—interacts with the AP-1 complex and is regulated by extracellular molecules that are involved in (dopaminergic) apoptosis, such as CRH, TAC1, and VEGFA. The third important signaling cascade in the nucleus centers around the transcription factors of the GADD45 family that regulate the neuronal stress response and apoptosis and interact with other transcription factors like CCND3 and CDKN1A. Further, most other landscape proteins interact with two proteins/protein complexes that regulate neuronal apoptosis and can be found both in the nucleus and cytoplasm, i.e., ERK1/2 and the adaptor protein STAT3. The signaling cascades dependent on ERK1/2 and STAT3 are in turn modulated by a number of extracellular proteins—e.g., FGF13, PLAT, and SCG2—and cytoplasmic regulators—e.g., DUSP14, HSPA5, and IRS2—with prominent roles in neuronal function and survival.

### qPCR Validation of Selected CREB1 Targets

To provide an additional validation of the RNA sequencing data, we performed qPCR to assess and compare the mRNA expression levels of 10 selected CREB1 targets. In Supplementary Fig. 4, the expression differences are shown for both comparisons (L-DOPA vs. saline and L-DOPA + Riluzole vs. L-DOPA). As we were able to validate 8 of the 10 tested mRNA expression differences and the two other differences were not statistically significant but still in the right direction, we submit that the RNA sequencing data, in general, and the expression data for the CREB1 targets, in particular, indeed reflect true expression changes.

## Discussion

In the current study, we ascertained that 6-OHDA-lesioned rats treated with L-DOPA demonstrate clear AIMs and this is counteracted by chronic exposure to Riluzole, a drug that has already been shown to have some effect on attenuating AIMs in previous studies. Our experiments revealed that Riluzole induces mRNA expression changes in the rat striatum that are tightly linked to the occurrence of L-DOPA-induced AIMs.

Our findings point towards the regulation of apoptosis as a key molecular mechanism underlying both the effect of chronic L-DOPA administration—with the accompanying AIMs—and the “rebalancing” effect of Riluzole, leading to a reduction of AIMs. In general terms, the landscape that we built based on the CREB1 targets that were expressed in the opposite direction in the AIMs model and after Riluzole administration suggests that chronic administration of L-DOPA tilts the neuronal survival/apoptosis balance towards increased survival (and, hence, less apoptosis). Conversely, Riluzole administration leads to a reversal of this balance towards less survival and more apoptosis. Indeed, most of the landscape genes encode proteins that have anti-apoptotic properties, and these genes are upregulated after chronic L-DOPA administration, an effect that is counteracted by Riluzole. Mechanistically, as derived from the upstream regulator analysis, the effect of Riluzole is mediated through decreasing the activation of CREB1, i.e., CREB1 (and, hence, the regulation of its downstream targets) is predicted to be activated upon exposure to L-DOPA and inhibited after adding Riluzole. CREB1 acts as a functional “go-between” between cytoplasmic kinase/enzyme signaling cascades and nuclear regulation of gene expression. More specifically, CREB1 is known to be activated through phosphorylation by kinases, such as ERK1/2 [44] and protein kinase C (PKC) [45] (see subsequent texts), which in turn leads to reduced neuronal apoptosis through the upregulation of anti-apoptotic gene expression by activated CREB1 [46]. In addition, L-DOPA administration to 6-OHDA-lesioned rats was found to markedly increase CREB1 phosphorylation in striatal neurons [47, 48], and, interestingly, a very recent study showed that Riluzole reduces neuronal CREB1 phosphorylation [49].

**Fig. 4:**
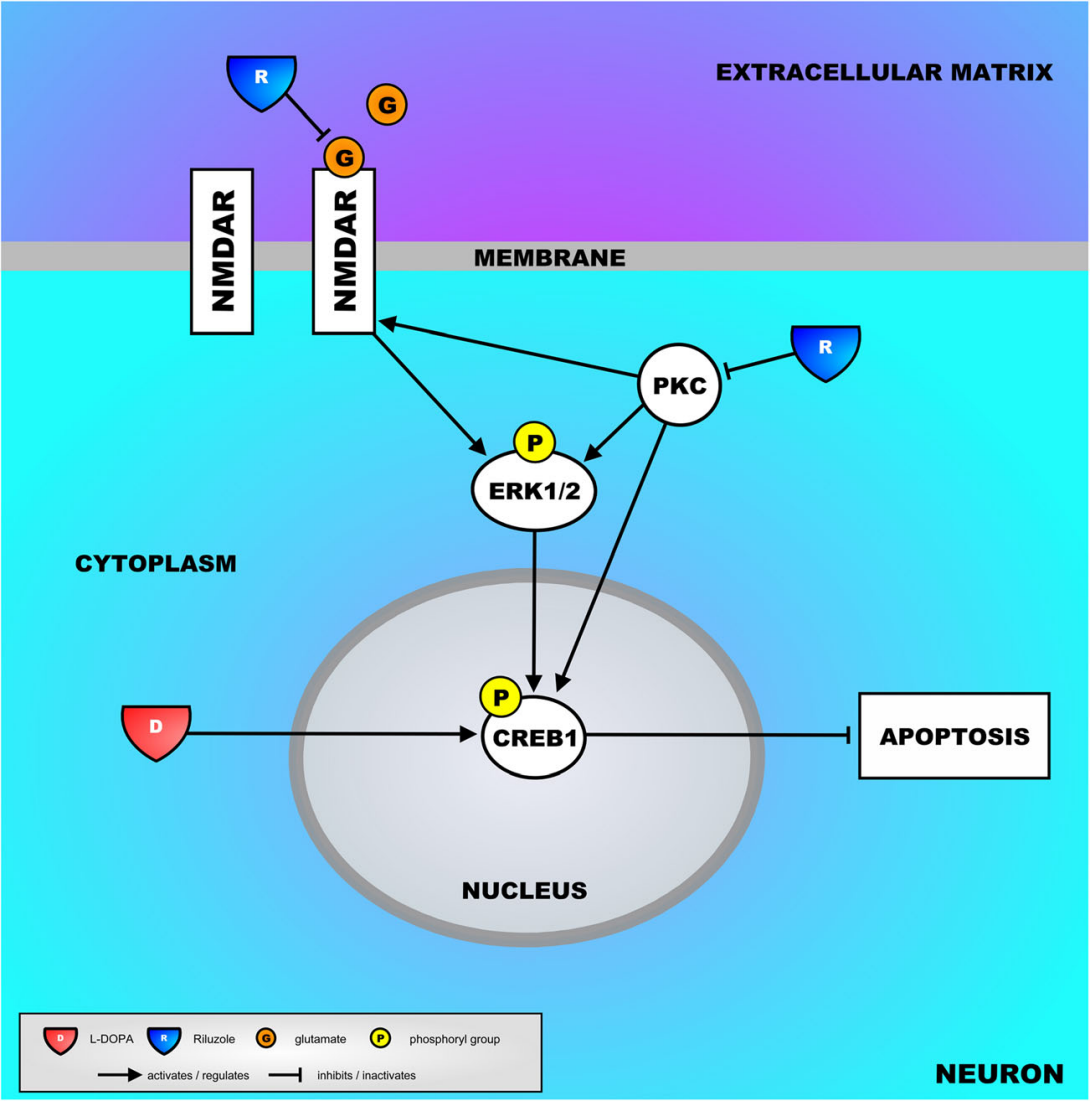
Putative mechanisms of how Riluzole could counteract CREB1-mediated gene expression by decreasing the activity of CREB1. Firstly, through a number of mechanisms, Riluzole reduces glutamatergic neurotransmission, which includes less glutamate binding to post-synaptic NMDA glutamate receptors. As a result, NMDA-mediated ERK1/2 signaling and the subsequent activation of CREB1 are also reduced. Secondly, Riluzole could inhibit PKC, a kinase that positively regulates NMDA receptors and activates CREB1 (directly or through activating ERK1/2), leading to a reduced CREB1 activity. The key players in this mechanism—L-DOPA, NMDA glutamate receptors, the kinases ERK1/2 and PKC, and CREB1 itself—are shown

As for how Riluzole would decrease CREB1 activity, there are some clues from the literature. Taken together with our results, these literature findings have led us to propose a putative molecular mechanism—shown in Fig. 4—through which Riluzole could decrease CREB1 activity, modulate apoptosis, and ultimately reduce AIMs. First, and as pointed out previously, Riluzole mainly exerts anti-glutamatergic effects which, through a number of mechanisms [27–31], lead to reduced glutamatergic neurotransmission. When activated by glutamate, post-synaptic NMDA receptors activate ERK1/2, kinases with an important role in our landscape that subsequently phosphorylate—and, hence, activate—CREB1 [44]. These data imply that through its anti-glutamatergic effects, Riluzole would decrease CREB1 activity by reducing NMDA receptor-induced ERK1/2 signaling. Second, Riluzole is a potent intracellular inhibitor of PKC [50], a kinase that positively regulates NMDA receptor function [51] and phosphorylates/activates CREB1, either directly or through activating ERK1/2 [45]. Although not shown in Fig. 4, a recent paper reported that Riluzole also reduces presynaptic glutamatergic vesicle recycling and, hence, the size of the readily releaseable glutamate pool through inhibiting PKC [31]. Further, it is interesting that glutamate-induced activation of CREB1 is (partially) blocked by inhibiting ERK1/2 with U0126 [52], a compound that, as opposed to CREB1, is predicted to be inhibited after exposure to L-DOPA and activated by adding Riluzole (see Supplementary Table 2).

Although our study yielded interesting findings, it has some limitations. First, while chronic L-DOPA administration is thought to be the main contributor to the AIMs phenotype through its effect on striatal projection neurons—i.e., through promoting dopamine receptor hypersensitivity in these neurons—lesioning with 6-OHDA affects dopaminergic as well as noradrenergic neurons. As such, a contribution of 6-OHDA-induced noradrenergic denervation to the AIMs phenotype cannot be excluded. In addition, although our results indicate that the effect of Riluzole on reducing AIMs is mediated through lowering CREB1 activity, further studies are needed to confirm that Riluzole (in)directly decreases CREB1 activity through reducing its phosphorylation. In this respect, it is interesting to note that the literature provides some corroborating evidence that phosphorylation-dependent CREB1 activation is indeed an important contributing factor to the development of AIMs. For example, psychostimulants, such as amphetamines and cocaine, and other drugs, such as the anti-psychotic aripiprazole—that are all known to induce AIMs [53–55]—promote phosphorylation-dependent CREB1 activation in (striatal) neurons [56–58]. Therefore, in addition to confirming the effect of Riluzole, future studies could test other (novel or existing) drugs for their positive effect on AIMs (and LID) through decreasing CREB1 activity.

In conclusion, we have demonstrated that Riluzole attenuates AIMs in 6-OHDA-lesioned rats that were chronically treated with L-DOPA. In addition, RNA sequencing analysis revealed that Riluzole reverses the expression direction of genes regulating mainly pro-apoptotic processes, downstream of activated CREB1. This molecular mechanism underlying the beneficial effect of Riluzole needs to be confirmed in future studies and can be leveraged to design AIMs/LID treatment studies using novel and/or existing compounds.

## Electronic supplementary material

ESM 1 (DOCX 478 kb)

ESM 2 (XLSX 429 kb)
