# Supplementary material for: Riluzole Attenuates L-DOPA-Induced Abnormal Involuntary Movements Through Decreasing CREB1 Activity: Insights from a Rat Model
Source: Mol Neurobiol. 2018 Nov 27;56(7):5111–21. doi: 10.1007/s12035-018-1433-x (PMC6647536; doi:10.1007/s12035-018-1433-x)
Supplement: Supplementary file 1 — (DOCX 478 kb) [file 12035_2018_1433_MOESM1_ESM.docx]

**Supplementary Materials**

*Molecular Neurobiology*

Riluzole attenuates L-DOPA-induced abnormal involuntary movements through decreasing CREB1 activity: insights from a rat model

Luca Pagliaroli^†^, Joanna Widomska^†^, Ester Nespoli, Tobias Hildebrandt, Csaba Barta, Jeffrey Glennon, Bastian Hengerer, Geert Poelmans^*^

^†^ Equal contribution

^*^ **Corresponding author**:

Geert Poelmans, M.D., Ph.D.

Department of Human Genetics, Radboud University Medical Center, Nijmegen, The Netherlands

Email: [geert.poelmans@radboudumc.nl](mailto:geert.poelmans@radboudumc.nl)

Supplementary Methods

**Stereotaxic surgery**

After 1 week of habituation at post-natal day (PD) 21 (n=48), rats were separated from their mothers and were stereotaxically injected with a 4.4 µg/µl solution of 6-hydroxydopamine hydrobromide (6-OHDA, Sigma-Aldrich, Germany) in 0.02% ascorbate solution (4µl of injected volume at a speed of 0.3 µL/min) into the left medial forebrain bundle (MFB) according to the following coordinates calculated (in cm) from bregma: Anterior Posterior= -0.41, Medial Lateral= + 0.15, Dorsal Ventral= -0.75. After injection, the needle was left in place for 3 additional minutes and then retracted. Surgical anesthesia was induced with 4.5% isoflurane in N_2_O/O_2_ (70: 30) and reduced to a minimum of 1.2-1.5% isoflurane to ensure anesthesia maintenance. Analgesia was provided with Meloxicam (1mg/kg SC in 2mg/ml injection volume, Boehringer Ingelheim Vetmedica GmbH, Germany) 20 minutes before the surgical procedure took place and at its end. After recovery, rats were housed in groups of 5 siblings to prevent isolation.

**Striatal DA determination through HPLC**

DA levels in 12 6-OHDA-lesioned striata compared to their contralateral controls were quantified using HPLC coupled to ECD. Frozen samples were homogenized by sonication in a solution of 0.4 N perchloric acid and centrifuged for 20 min at 4 °C. Supernatants were filtered and used for DA quantification. The HPLC system was composed of an HPLC P680 ISO isocratic pump system and an automated ASI-100 sample injector (Dionex, Dreieich, Germany). The electrochemical detector was set at the potential of +650 mV using a glassy carbon electrode and an Ag/AgCl reference electrode (Antec VT-03, Zoeterwoude, Netherlands). A reversed-phase column (100 x 2.1 mm i.d. with pre-column 10 x 2.1 mm i.d., filled with ODS-AQ, 120 Å, 3 µm, YMC Europe GmbH, Dinslaken, Germany) was used for chromatographic separation. The mobile phase consisted of 1.7 mM of 1-octanesulfonic acid sodium salt, 1.0 mM Na2EDTA x 2 H2O, 8.0 mMNaCl, 100.0 mM NaH2PO4 x 2 H2O, adjusted to pH 3.80 with H3PO4, filtered through a 0.22 µm filter (Merck Millipore, Darmstadt, Germany), mixed up with 9.3 % acetonitrile, and delivered with a flow rate of 0.4 ml/min. Aliquots of 10uL were injected by an autosampler with a cooling module temperature of 4 °C (ASI-100, Thermo Scientific Dionex, Dreieich, Germany). DA was quantified through the comparison with an external five-point standard calibration. Chromeleon™ 7.1 software (Thermo Scientific Dionex, Dreieich, Germany) was used for data acquisition and calculation.

Supplementary Materials - Description of the molecular landscape

As described in the Methods section, we built a molecular landscape located in a neuron that is based on a subset of genes/mRNAs that were differentially expressed between three groups of rats and are all downstream expression targets of CREB1 (cAMP response element binding protein 1). All these genes are listed in Supplementary Data file 1 and in the description below; the proteins encoded by these genes are indicated in bold. Moreover, the evidence linking a number of these genes/proteins to L-DOPA signaling is presented.

Signaling in the molecular landscape originates in the nucleus and is centered around CREB1. As main signaling 'hub' in the landscape, CREB1 is an important transcription factor that regulates the expression of a large number of target genes involved in many neuronal processes, including neuronal survival, differentiation and development. L-DOPA has been found to directly activate the transcriptional function of CREB1 in striatal neurons [1,2]. Another important member of the CREB transcription factor family is **CREM** [3] which binds [4] and is involved in regulating the expression of CREB1 [5]. The CREB1-**CREM** complex also binds and interacts with **FOS** and **JUN** [6]. **FOS** and **JUN** bind each other as well as **FOSB** and **JUNB**. **FOS**, **JUN**, **FOSB** and **JUNB** are part of the AP-1 complex of transcription factors that interacts with CREB1 [7-11] and is known to play a role in apoptosis of (neuronal) cells in response to stress, DNA damaging agents and a general lack of 'survival signals' [12]. Further, CREB1 and **CREM** inhibit the transcriptional activity of **JUN** [6]. In addition, **FOS** and **JUN** interact with **ATF3** [13] and **CREM** interacts with the transcription factor **NFIL3** [14] (see below). **JUN** regulates neuronal differentiation and cell survival. More specifically, **JUN** protects against apoptosis in differentiated PC12 cells that are often used as a model of neuronal differentiation [15]. Interestingly, increased expression levels of **Fos** were reported in rodents that had been injected with 6-hydroxy-dopamine (6-OHDA) [16,17] and subsequently treated with L-DOPA [18-20]. L-DOPA administration also produces a long-lasting increase in the expression levels of **FosB** but not **JunB** [21]. In keeping with these finding, **JunB** was shown to protect against the death of nigral neurons [22].

Furthermore, the extracellular signal-regulated kinases 1 and 2 (ERK1/2) - that can function in both the cytoplasm and nucleus - activate both CREB1 and **CREM** (not shown) [23,24]. In addition, ERK1/2 are involved in increasing the expression of **FOSB** [25] and activate **FOS** [26,27] and **ATF3** [28]. ERK1/2 were originally identified as kinases that regulate neuronal survival and neuroprotection but subsequently, it was found that ERK1/2 also play a critical role in secondary damage mechanisms implicated in a number of neurodegenerative diseases, stroke, CNS injury, and autoimmune diseases of the CNS [29-34]. In addition, a number of studies reported that activation of ERK1/2 in the dopamine-depleted striatum is induced by L-DOPA administration [35-39]. **ATF3** and ERK1/2 are involved in decreasing the expression of **ID1** [40,41], an anti-apoptotic transcription factor [42], while **ID1** decreases the expression of ERK1/2 [43]. Further, under normal circumstances, **ATF3** is expressed at low levels in both neurons and glia cells but its expression is upregulated in response to injury or stressful stimuli, after which it positively regulates the survival of these cells [44]. In line with our findings, a chromatin immunoprecipitation (ChIP) study also showed that in 6-OHDA-lesioned striata of mice, the expression of **Atf3** as well as the transcription factors **Klf4** and **Npas4** (see below) was upregulated after acute L-DOPA administration [45].

Moreover, **NR4A1** and **NR4A3** belong to the NR4A orphan nuclear receptor family of transcription factors that is rapidly and strongly upregulated after stressful insults to the CNS. NR4A transcription factors are essential for neuronal survival downstream of CREB(1) signaling and may be suitable targets for intervention in neurodegenerative disease [46].

In the MPTP mouse model of PD, **Nr4a1** activation protects dopaminergic neurons [47]. In addition, ***Nr4a1*** knockout mice display higher locomotor activity, a greater sensitivity to dopamine [48] and increased L-DOPA-induced rotational behaviour [49]. Further, **Nr4a1** expression was increased in the caudate nucleus and putamen upon L-DOPA treatment in MPTP monkeys (that are a non-human primate model of PD) [50]. Lastly, ***NR4A1*** expression was found to be decreased in the blood of PD patients [51]. All these findings are in line with our results showing an overexpression of **NR4A1**. Within the landscape, **NR4A1** binds and forms a functional complex with **NR4A3** [52], **NR4A1** decreases the activity of **FOSB** [53] and the kinase **SIK1** - which can be found in both the nucleus and cytoplasm and is upregulated by **CREM** [54] - reduces **NR4A1** expression [55]. In addition, **TAC1 -** an extracellular, potently anti-apoptotic protein [56] that was also found to be increased in a primate model of L-DOPA induced dyskinesia [57] - activates **NR4A1** [58] and upregulates the expression of **FOSB** [59], which apart from the nucleus can also be found in the cytoplasm, were it forms a functional complex with **BAG3**, a co-chaperone protein that regulates neuronal apoptosis and autophagy [60,61]. Interestingly, **Bag3**-mediated autophagy is also involved in the clearance of aggregated proteins associated with age-related neurodegenerative disorders [62-64]. Lastly, corticotropin-releasing hormone (**CRH**) - which functions as a neurotransmitter in the central nervous system (CNS) [65] and regulates neuronal apoptosis [66] as well as dopaminergic neuron function [67] - increases the expression of **JUNB** [68] while **FOSB** increases the expression of **CRH** [69]. Further, **CRH** upregulates the expression of **NR4A1** [70], **TAC1** [71] and the growth factor (see below) **VEGFA** [72] and it activates ERK1/2 [73]. In turn, ERK1/2 upregulates **NR4A3** [74].

Another signaling cascade in the landscape centers around **CDKN1A**, a transcription factor that is also known as p21 (WAF1/CIP1) and is involved in 6-OHDA-induced dopaminergic cell death [75]. **CDKN1A** inhibits **FOS** and **JUN** [76] while it activates ERK1/2 [77]. **KLF4** and ERK1/2 are involved in upregulating **CDKN1A** expression [77,78], while ERK1/2 also increases the expression of **KLF4** [79]. Further, the growth factor **FGF13** (see below), the transcription factor **SERTAD1** and the cytoplasmic protein **FRMD6** all increase the expression of **CDKN1A** [80-82], whereas **ID1** and **BAG3** decrease its expression [83,84]. **CDKN1A** also inhibits **CCND3** [85], a nuclear regulator of the cell cycle that was found to be significantly increased during 6-OHDA-induced apoptosis of dopaminergic neurons, which is interesting given that loss of cell cycle control has been suggested to lead to death of dopaminergic neurons observed in Parkinson's disease (PD) [86]. Moreover, **GADD45G** - a member of the GADD45 family of nuclear proteins that have an important role in regulating the neuronal stress response and apoptosis [87] - binds, interacts with, and increases the expression of **CDKN1A** [88] and also binds/interacts with **MIDN** [89], a nuclear protein encoded by a gene that has been strongly linked to Parkinson's disease [90,91]. **GADD45G**, **GADD45A** and **GADD45B** - which also upregulates **CDKN1A** expression [92] - bind and form a functional complex [93]. Interestingly, knockout of ***Gadd45b*** in the L-DOPA-induced dyskinesia (LID)-rodent model results in an increased expression of **Fos** and **FosB** [94], which implies that **GADD45B** downregulates the expression of **FOS** and **FOSB** (not shown). Another study on LID showed that the expression of **Gadd45b** and **Gadd45g** is increased in the lesioned side of the striatum after L-DOPA administration [95]. **Gadd45a** is also involved in 6-OHDA-induced brain toxicity in rats [96]. **NFIL3 -** a transcription factor that plays a neuroprotective role in neurons and constitutes a potential therapeutic target for neurodegeneration [97] - also downregulates the expression of **GADD45B** [98].

Multiple landscape proteins interact with **STAT3**, a protein that can function in both the cytoplasm and nucleus. **STAT3**-dependent signaling plays an important role in counteracting 6-OHDA-induced neuronal cell death by promoting compensatory neuronal proliferation [99]. In the nucleus, **STAT3** is involved in downregulating the expression of **GADD45B** and **GADD45G** [100,101], while it is inhibited by **GADD45A** [102] and **KLF4** [103]. Further, **STAT3** activates **CDKN1A** [104], whereas **CDKN1A** inhibits **STAT3** [105]. In the cytoplasm, **STAT3** forms a functional complex with **HSPA5**, which in turn binds and interacts with **CDC37** and **BAG3** [106,107]. **HSPA5** and **CDC37** are regulators of normal protein homeostasis and the unfolded protein response under physiological stimulation [108-110]. Moreover, it has been reported that increased levels of **Hspa5** protein protect against dopaminergic neurodegeneration in a rat model of PD [111]. ERK1/2 also upregulates **HSPA5** expression [112] while **BAG3** inhibits ERK1/2 function [113]. In addition, **STAT3** and ERK1/2 activate each other [114,115]. **STAT3** is involved in upregulating the expression of **VEGFA** [116] (see below), while the extracellular, neuroendocrine protein **secretogranin-2** **(SCG2)** activates **STAT3** [114]. **SCG2** is the precursor of secretoneurin (SN) (not shown), which modulates neurotransmission and inflammatory responses and is involved in neuronal differentiation [117]. Moreover, it was shown that SN induces expression of anti-apoptotic proteins through **STAT3**-dependent signaling [114]. ERK1/2 regulates the expression of **SCG2** [118] and its activity is regulated by the **FOS-JUN**-complex [117]. This complex also upregulates **SRXN1** [119], a cytoplasmic enzyme that protects against oxidative stress-induced damage of dopaminergic neurons [120,121].

There are a number of other signaling cascades in the landscape involving **ERK1/2**. First, **TAC1** (see above) activates ERK1/2 [122], while ERK1/2 is involved in upregulating the expression of both **TAC1** [123] and **PTGS2** [124], a cytoplasmic enzyme that produces prostaglandin, and is upregulated through signaling involving the extracellular proteins **CRH** [125] and **VEGFA** [126] (see above) and downregulated by **NFIL3** (see above) [127]. Furthermore, ERK1/2 regulates the activity of **NPTX2** [128,129], an extracellular protein that is upregulated by **NPAS4** [130]. **NPTX2** is important in neuronal development, neuronal migration, synapse formation, and neurite outgrowth [131,132]. In addition, **Nptx2** was found to be extremely upregulated (>800%) in the substantia nigra of PD patients [131] and it was reported that **Nptx2** expression increases upon L-DOPA administration while LID severity is reduced in ***Nptx2*** knockout mice [129]. Further, ERK1/2 activity is regulated by the cytoplasmic adaptor protein **SH3KBP1** and by **IRS2** [133], a cytoplasmic insulin-signaling related protein that is also upregulated downstream of ERK1/2 [123]. **IRS2** is implicated in apoptosis and in the regulation of the dopaminergic cell morphology [134], and 6-OHDA injection leads to degradation of **Irs2** [135]. Moreover, the GTP-binding protein **RHEB** inhibits ERK1/2 [136] and decreases the expression of **IRS2** [137]. **RHEB** regulates neuronal plasticity and differentiation in response to injury and stress, and it was shown to preserve and restore nigrostriatal dopaminergic axonal projections in a PD mouse model [138-140]. Interestingly, when it is overexpressed, **Rheb** is able to switch its function and hence become an apoptotic enhancer. ERK1/2 also upregulates the expression of **EGR4**, a transcription factor that is increased by L-DOPA [129]. Another negative regulator of ERK1/2 function is the phosphatase **DUSP14** [141,142] and as such, it negatively regulates ERK signaling in dopamine-depleted striatal neurons during LID [143,144]. **DUSP14** is also involved in downregulating te expression of **PLAT** [145], an extracellular enzyme with roles in neuronal migration and plasticity [146]. In vitro and ex vivo studies also suggest that **Plat** has pro-survival/anti-apoptotic effects on both neurons and oligodendrocytes [147]. **PLAT** increases the expression of the cytokine **INHBA** [148], and both **PLAT** and **INHBA** activate **ERK1/2** [149,150]. In addition, **INHBA** increases the expression of **JUNB** [151] and **VEGFA** [150], a growth factor that itself activates **JUNB** [152] and ERK1/2 [153], and is upregulated downstream of ERK1/2 [154] and **FGF13** [155], a growth factor that also activates **JUNB** [8]. **VEGFA** has a key role in vascular and neuronal pattering in the developing central nervous system (CNS) [156] and **VEGFA** administration to 6-OHDA- lesioned rats lead to a significant increase in neuronal number [157]. Further, **VEGFA** increases the expression of **FOSB** [158] and **NR4A3** [159] (see above), while **VEGFA**, **TAC1** and **KLF4** all upregulate **PLAT** expression [160-162]. **CRH** activates **PLAT** [163] and is activated by **NPY**, an extracellular hormone that is abundant in the CNS where it negatively and positively regulates apoptosis and autophagy, respectively [164,165], and that activates ERK1/2 [166]. **NPY** expression is also increased in the striatum of PD patients where it acts as a neuroprotective agent [167]. Further, **CRH** expression is downregulated by the kinase **SIK1** [168] (see above) that also upregulates the expression of **ARC**, a brain-specific protein with a role in long-term synaptic plasticity and apoptosis [169,170] that was upregulated in the striatal neurons of a rat LID-model [171,172]. In addition, **ARC** is activated by ERK1/2 [173] and downregulated by **PDYN** [174], a neuropeptide hormone that is upregulated by **TAC1** [59] and has anti-apoptotic effects [175]. Increased expression of **Pdyn** was also observed in the striatum of a rodent LID model [176-178]. Lastly, ERK1/2 upregulates the expression of **PER1** [179], a circadian clock protein that shuttles between the nucleus and cytoplasm and is upregulated by **FGF13** [180], and - in the nucleus - inhibited by **NFIL3** [3] (not shown). **FGF13** is widely expressed in the developing brain and it has a key role in establishing neuronal circuits in the cerebral cortex as well as neuronal polarization [181]. Furthermore, **FGF13** activates ERK1/2 [182].

**Supplementary Fig. 1.** Effect of unilateral 6-OHDA lesion in the MFB and chronic L-DOPA treatment on rat striatal tissue DA levels measured in the same animals (n= 12). Data is shown as mean +/- S.E.M with **** denoting p<0.001.


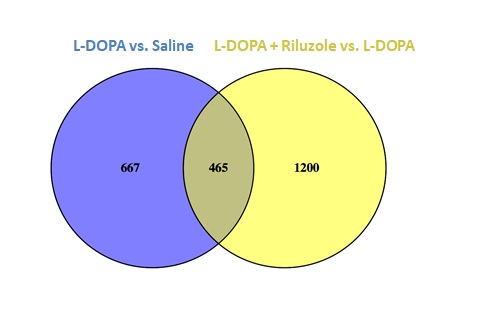


**Supplementary Fig. 2.** Venn diagram of unique and overlapping significantly differentially expressed genes (FDR corrected p-value <0.01) in comparisons L-DOPA vs. Saline and L-DOPA+Riluzole vs. L-DOPA. All of these genes were differentially expressed in the opposite direction in the two comparisons. Using the hypergeometric test, we determined that this overlap in differentially expressed genes is highly significant (hypergeometric p-value = 1.01E-142).


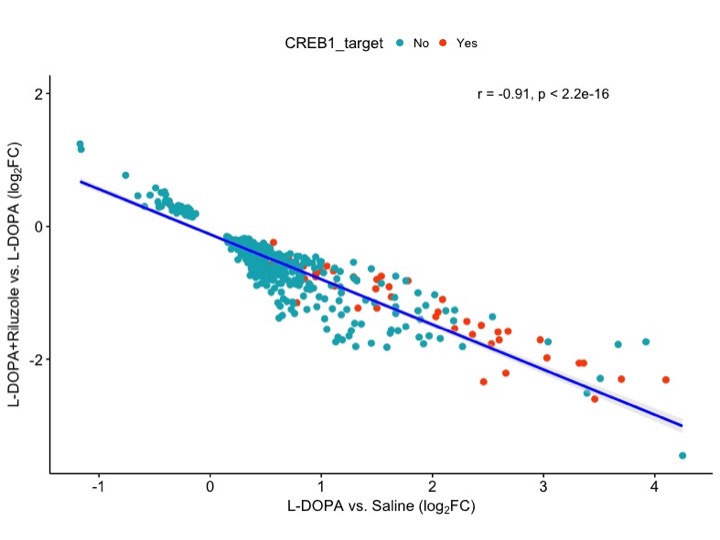


**Supplementary Fig. 3.** Scatter plot representing the correlation of log_2_ fold changes observed for overlapping differentially expressed genes - including 58 direct CREB1 target genes - in both comparisons.


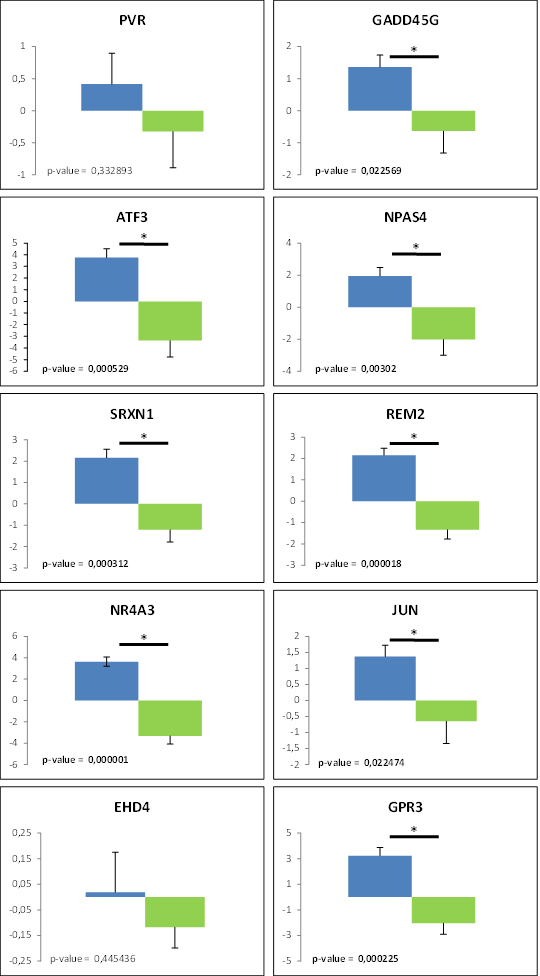


**Supplementary Fig. 4.** qPCR validation of 10 selected CREB1 targets. The housekeeping genes *Bcap29* and *Cdkn1b* were used as reference for normalization of gene expression, and a Student’s t-test was used to assess statistical significance. Values are shown as log_2_FC (Mean + SEM) with blue and green bars representing L-DOPA vs. Saline and L-DOPA+Riluzole vs. L-DOPA, respectively.

**Supplementary Table 1.** List of primers used for qPCR validation

| **RT² qPCR Primer Assay (Qiagen)** | | |
| --- | --- | --- |
| Gene Symbol | Catalog number | RefSeq Accession |
| *Jun* | PPR53221A | NM_021835.3 |
| *Npas4* | PPR52619A | NM_153626.1 |
| *Gpr3* | PPR52464A | NM_153727.1 |
| *Nr4a3* | PPR51343E | NM_017352.1 |
| *Srxn1* | PPR50360B | NM_00147858.3 |
| *Gadd45g* | PPR46380A | NM_001077640.1 |
| *Pvr* | PPR44991A | NM_017076.2 |
| *Atf3* | PPR44403B | NM_012912.2 |
| *Rem2* | PPR06549B | NM_022685 |
| *Ehd4* | PPR43831A | NM_139324 |
| **Housekeeping genes** | | |
| Gene Symbol | Forward Primer | Reverse Primer |
| *Bcap29* | AGAAGGCTTCCGATGCCCT | TGCTCTTTCAGGAGTCGGTCA |
| *Cdkn1b* | CAGACGTAAACAGCTCCGAATT | CTCAGTGCTTATACAGGATGTC |

**Supplementary Table 2.** Top 10 upstream regulators of significantly differentially expressed genes in comparisons L-DOPA vs. Saline and L-DOPA+Riluzole vs. L-DOPA (FDR corrected p-value <0.01) revealed by Upstream Regulator Analysis performed in IPA.

| Upstream Regulator | Type | Activation z-score | P-value of overlap | # Target genes |
| --- | --- | --- | --- | --- |
| L-DOPA vs. Saline | | | | |
| CREB1 | transcription regulator | 7,52 | 8,53E-31 | 102 |
| Forskolin | chemical toxicant | 6,06 | 5,56E-23 | 100 |
| Cocaine | chemical drug | 5,58 | 1,02E-19 | 48 |
| PDGF BB | Complex | 5,22 | 1,74E-19 | 61 |
| beta-estradiol | chemical - endogenous mammalian | 4,05 | 2,01E-19 | 178 |
| U0126 | chemical - kinase inhibitor | -5,07 | 7,54E-19 | 76 |
| CREM | transcription regulator | 4,19 | 7,25E-18 | 39 |
| dalfampridine | chemical drug | 4,58 | 2,66E-17 | 21 |
| TGFB1 | growth factor | 3,82 | 1,10E-16 | 166 |
| bicuculline | chemical - endogenous non-mammalian | 4,48 | 7,07E-15 | 21 |
| L-DOPA+Riluzole vs. L-DOPA | | | | |
| CREB1 | transcription regulator | -6,53 | 1,21E-11 | 87 |
| F2 | Peptidase | -6,33 | 8,46E-09 | 48 |
| TP53 | transcription regulator | -2,80 | 2,41E-08 | 167 |
| CREM | transcription regulator | -2,73 | 3,16E-08 | 32 |
| 2-amino-5-phosphonovaleric acid | chemical - other | 3,20 | 3,57E-08 | 31 |
| PDGF BB | Complex | -5,31 | 6,20E-08 | 52 |
| Forskolin | chemical toxicant | -5,48 | 1,26E-07 | 87 |
| HIF1A | transcription regulator | -4,27 | 2,98E-07 | 58 |
| CD40LG | Cytokine | -2,50 | 3,17E-07 | 57 |
| U0126 | chemical - kinase inhibitor | 4,82 | 4,30E-07 | 67 |
| Overlapping genes (FC for Riluzole+L-DOPA vs. L-DOPA) | | | | |
| CREB1 | transcription regulator | -6,19 | 1,17E-24 | 58 |
| Forskolin | chemical toxicant | -5,88 | 5,08E-22 | 60 |
| Cocaine | chemical drug | -4,32 | 1,71E-15 | 28 |
| CREM | transcription regulator | -3,00 | 5,60E-15 | 24 |
| dalfampridine | chemical drug | -3,74 | 2,92E-14 | 14 |
| PDGF BB | complex | -4,98 | 3,68E-14 | 33 |
| U0126 | chemical - kinase inhibitor | 4,57 | 4,27E-13 | 39 |
| kainic acid | chemical toxicant | -4,33 | 7,23E-13 | 22 |
| bicuculline | chemical - endogenous non-mammalian | -3,65 | 8,91E-13 | 14 |
| IL1B | cytokine | -3,60 | 1,59E-12 | 54 |

**Supplementary Table 3.** Gene enrichment analysis of significantly differentially expressed genes (FDR corrected p-value <0.01) in comparisons L-DOPA vs. Saline and L-DOPA+Riluzole vs. L-DOPA.

| **Diseases and Disorders** | | | **Molecular and Cellular Functions** | | |
| --- | --- | --- | --- | --- | --- |
| Category | P-value of overlap | # Genes | Category | P-value of overlap | # Genes |
| **L-DOPA vs. Saline** | | | | | |
| Cancer | 9,65E-27 | 979 | Cell death | 6,63E-17 | 387 |
| Epileptic seizure | 4,95E-26 | 60 | Morphology of cells | 4,95E-14 | 257 |
| Disorder of basal ganglia | 1,83E-18 | 134 | Expression of RNA | 5,15E-11 | 241 |
| Neuromuscular disease | 3,52E-16 | 142 | Differentiation of connective tissue cells | 2,05E-09 | 96 |
| Huntington's Disease | 1,38E-13 | 96 | Organization of cytoplasm | 2,68E-09 | 182 |
| Proliferation of tumor cells | 6,68E-08 | 71 | Cell cycle progression | 3,94E-09 | 135 |
| Hereditary optic atrophy | 1,04E-06 | 11 | Cell proliferation of fibroblasts | 5,28E-09 | 60 |
| Abnormal morphology of cardiovascular system | 1,08E-06 | 73 | Cell movement | 1,23E-08 | 243 |
| Abnormal morphology of skin | 4,05E-05 | 36 | Metabolism of protein | 2,62E-08 | 124 |
| Inflammation of joint | 9,70E-05 | 115 | Transport of molecule | 6,12E-06 | 177 |
| **L-DOPA+Riluzole vs. L-DOPA** | | | | | |
| Cancer | 5,86E-38 | 1403 | Transcription of RNA | 2,15E-19 | 310 |
| Liver lesion | 9,54E-10 | 605 | Morphology of cells | 1,11E-15 | 349 |
| Growth failure | 1,30E-09 | 112 | Organization of cytoplasm | 1,92E-14 | 263 |
| Epileptic seizure | 1,10E-08 | 44 | Differentiation of connective tissue cells | 8,70E-12 | 131 |
| Infection of cells | 7,22E-07 | 127 | Cell death | 1,92E-11 | 494 |
| Abnormal morphology of cardiovascular system | 7,76E-07 | 95 | Development of neurons | 1,13E-09 | 144 |
| Proliferation of tumor cells | 1,46E-05 | 83 | Cell movement | 1,50E-09 | 333 |
| Pancreatic mass | 2,92E-05 | 335 | Cell cycle progression | 5,30E-07 | 168 |
| Autosomal dominant disease | 3,67E-04 | 126 | Protein kinase cascade | 2,93E-05 | 69 |
| Phagocytosis of tumor cell lines | 5,67E-04 | 19 | Metabolism of protein | 1,66E-04 | 145 |
| **Overlapping genes** | | | | | |
| Epileptic seizure | 2,00E-21 | 38 | Transcription of RNA | 8,48E-15 | 117 |
| Cancer | 1,06E-11 | 400 | Apoptosis | 1,62E-11 | 146 |
| Proliferation of tumor cells | 2,16E-08 | 41 | Differentiation of connective tissue cells | 2,37E-11 | 57 |
| Necrosis of muscle | 3,52E-05 | 28 | Binding of DNA | 5,71E-11 | 51 |
| Abnormal morphology of embryonic tissue | 6,35E-05 | 36 | Morphology of cells | 1,99E-09 | 118 |
| Cell death of liver cells | 1,65E-04 | 19 | Colony formation | 2,19E-09 | 47 |
| Necrosis of kidney | 4,28E-04 | 26 | Cell movement | 3,54E-07 | 114 |
| Inflammation of organ | 4,72E-04 | 67 | Formation of filaments | 8,37E-07 | 34 |
| Inflammation of joint | 1,93E-03 | 52 | Lymphocyte homeostasis | 8,75E-06 | 40 |
| Abnormal morphology of skin | 5,77E-03 | 16 | Synthesis of protein | 1,41E-04 | 32 |

**Supplementary Table 4.** Top 10 'Diseases and Disorders' and 'Molecular and Cellular Functions' categories revealed by gene enrichment analysis in IPA based on 58 overlapping target genes of CREB1. The genes in bold encode proteins that are included in our molecular landscape (**Fig. 3**).

| **Diseases and Disorders** | | |
| --- | --- | --- |
| Functions and Annotations | BH p-value | Genes |
| epileptic seizure | 2,98E-28 | ***Arc***, ***Atf3***, ***Bag3***, ***Cdkn1a***, ***Crem***, ***Egr4***, ***Fos***, ***Fosb***, ***Gadd45b***, ***Gadd45g***, ***Inhba***, ***Jun***, ***Junb***, ***Nfil3***, ***Nptx2***, ***Nr4a1***, ***Nr4a3***, ***Pdyn***, *Pim3*, ***Ptgs2***, ***Scg2***, ***Sertad1***, ***Tac1*** |
| proliferation of tumor cells | 1,67E-10 | ***Atf3***, ***Ccnd3***, ***Cdkn1a***, ***Crem***, ***Fos***, ***Hspa5***, ***Id1***, ***Inhba***, ***Jun***, ***Nr4a1***, *Pim3*, ***Plat***, ***Ptgs2***, ***Rheb***, ***Stat3***, ***Tac1***, ***Vegfa*** |
| weight gain | 9,67E-08 | ***Cdkn1a***, ***Crh***, ***Fos***, ***Id1***, ***Inhba***, ***Junb***, ***Npy***, ***Nr4a1***, ***Ptgs2***, *Slc32a1*, ***Stat3***, ***Vegfa*** |
| hypertrophy of heart | 1,34E-07 | ***Atf3***, ***Cdkn1a***, ***Crem***, *Gaa*, ***Inhba***, ***Jun***, ***Klf4***, ***Nr4a3***, ***Plat***, ***Ptgs2***, ***Rheb***, ***Stat3*** |
| inflammation of organ | 1,34E-07 | ***Atf3***, ***Cdkn1a***, ***Crh***, ***Dusp14*,** *Gaa*, ***Gadd45b***, ***Gadd45g***, ***Hspa5***, ***Id1***, ***Jun***, ***Junb***, ***Klf4***, ***Nfil3***, ***Npy***, ***Nr4a1***, ***Pdyn***, ***Per1***, ***Plat***, ***Ptgs2***, ***Stat3***, ***Tac1***, ***Vegfa*** |
| T-cell lymphoproliferative disorder | 1,37E-06 | ***Ccnd3***, ***Cdkn1a***, ***Crem***, ***Gadd45b***, ***Id1***, ***Irs2***, ***Jun***, ***Lmo1***, ***Nr4a3***, ***Per1***, ***Sertad1***, ***Stat3***, ***Vegfa*** |
| Polyarthritis | 1,93E-06 | ***Fos****,* ***Fosb****,* ***Gadd45b****,* ***Nr4a1****,* ***Nr4a3****,* ***Plat****,* ***Ptgs2****,* ***Stat3*** |
| disorder of basal ganglia | 4,78E-06 | ***Crem***, ***Egr4***, ***Fgf13***, ***Fos***, ***Fosb***, ***Hspa5***, ***Jun***, ***Junb***, ***Npy***, ***Nr4a1***, ***Nrgn***, ***Ptgs2***, ***Scg2***, *Slc32a1*, ***Tac1*** |
| degranulation of mast cells | 6,53E-06 | ***Atf3***, ***Crh***, ***Fos***, ***Junb***, ***Nr4a3***, ***Plat***, ***Tac1*** |
| glucose metabolism disorder | 8,85E-06 | ***Atf3***, ***Bag3***, ***Cdkn1a***, ***Crem***, ***Crh***, ***Fos***, ***Gaa***, ***Hspa5***, ***Id1***, ***Irs2***, ***Jun***, ***Klf4***, ***Npy***, ***Nr4a1***, ***Nr4a3***, ***Pdyn***, ***Ptgs2***, ***Stat3***, ***Vegfa*** |
|  |  |  |
| **Molecular and Cellular Functions** | | |
| Functions and Annotations | BH p-value | Molecules |
| Apoptosis | 3,48E-17 | ***Arc***, ***Atf3***, ***Bag3***, ***Ccnd3***, ***Cdc37***, *Cdk19*, ***Cdkn1a***, ***Crem***, ***Crh*,** *Csrnp1*, ***Egr4*,** *Ehd4*, ***Fos***, ***Fosb***, ***Gadd45b***, ***Gadd45g***, ***Hspa5***, ***Id1***, ***Inhba***, ***Irs2***, ***Jun***, ***Junb***, ***Klf4***, ***Nfil3***, ***Npas4***, ***Nptx2***, ***Nr4a1***, ***Nr4a3*,** *Pdxk*, ***Pdyn***, ***Per1***, *Pim3*, ***Plat***, ***Ptgs2***, *Rem2*, ***Rheb***, *Sema7a*, ***Sh3kbp1***, ***Sik1***, ***Srxn1***, ***Stat3***, ***Tac1***, ***Vegfa*** |
| cell cycle progression | 1,03E-13 | ***Atf3***, ***Ccnd3***, ***Cdc37***, *Cdk19*, ***Cdkn1a***, ***Crh***, ***Fos***, ***Frmd6***, ***Gadd45b***, ***Gadd45g***, ***Id1***, ***Inhba***, ***Irs2***, ***Jun***, ***Junb***, ***Klf4***, ***Nr4a1***, ***Nr4a3***, ***Per1***, *Pim3*, ***Ptgs2***, *Rem2*, ***Sertad1***, ***Sik1***, ***Stat3***, ***Tac1***, ***Vegfa*** |
| colony formation of tumor cell lines | 2,56E-11 | ***Atf3***, ***Bag3***, ***Cdkn1a***, ***Fos***, ***Frmd6***, ***Gadd45b***, ***Gadd45g***, ***Id1***, ***Jun***, ***Klf4***, ***Nr4a1***, ***Ptgs2***, ***Sertad1***, ***Stat3***, ***Vegfa*** |
| proliferation of lymphocytes | 4,14E-11 | ***Ccnd3***, ***Cdkn1a***, ***Crh***, ***Dusp14***, ***Fos***, ***Gadd45b***, ***Hspa5***, ***Inhba***, ***Irs2***, ***Jun***, ***Junb***, ***Klf4***, ***Lmo1***, ***Nfil3***, ***Npy***, *Pim3*, ***Ptgs2***, ***Sh3kbp1***, ***Stat3***, ***Tac1***, ***Vegfa*** |
| transactivation of RNA | 8,18E-10 | ***Ccnd3***, ***Crem***, ***Fgf13***, ***Fos***, ***Fosb***, ***Gadd45g***, ***Id1***, ***Inhba***, ***Jun***, ***Junb***, ***Klf4***, ***Nr4a1***, ***Nr4a3***, ***Per1***, ***Ptgs2***, ***Sh3kbp1***, ***Stat3*** |
| cellular homeostasis | 2,6E-09 | ***Bag3***, ***Cdkn1a***, ***Crh***, ***Fos***, *Gaa*, ***Gadd45b***, ***Gadd45g***, *Gpr3*, ***Hspa5***, ***Id1***, ***Inhba***, ***Irs2***, ***Jun***, ***Junb***, ***Klf4***, *Lmo1*, ***Nfil3***, ***Npy***, ***Nr4a1***, ***Ptgs2***, ***Rheb***, ***Sh3kbp1***, ***Sik1***, ***Srxn1***, ***Stat3***, ***Tac1***, ***Vegfa*** |
| morphology of cells | 1,94E-08 | ***Atf3***, ***Ccnd3***, ***Cdkn1a***, ***Crem***, ***Crh***, ***Egr4***, *Ehd4*, ***Fgf13***, ***Fos***, *Gaa*, *Gpr3*, ***Id1***, ***Inhba***, ***Irs2***, ***Jun***, ***Junb***, ***Klf4***, ***Nfil3***, ***Npas4***, ***Npy***, ***Nr4a1***, ***Nr4a3***, *Pim3*, ***Plat***, ***Ptgs2***, ***Rheb***, ***Stat3***, ***Tac1***, ***Vegfa*** |
| degeneration of cells | 2,93E-08 | ***Atf3***, ***Crem***, ***Jun***, ***Nfil3***, ***Npas4***, ***Nr4a3***, ***Pdyn***, *Pim3*, ***Plat***, ***Ptgs2***, ***Rheb***, ***Stat3***, ***Vegfa*** |
| synthesis of DNA | 2,93E-08 | ***Atf3***, ***Cdkn1a***, ***Crem***, ***Fos***, ***Id1***, ***Inhba***, ***Irs2***, ***Jun***, ***Nr4a1***, ***Nr4a3***, ***Ptgs2***, ***Stat3***, ***Tac1***, ***Vegfa*** |
| incorporation of thymidine | 4,39E-08 | ***Atf3***, ***Cdkn1a***, ***Crem***, ***Inhba***, ***Jun***, ***Ptgs2***, ***Stat3***, ***Vegfa*** |

**References**

1. Kashihara K, Ishihara T, Akiyama K, Kuroda S, Morimasa T, Shomori T (1995) Levodopa induces AP-1 and CREB DNA-binding activities in the rat striatum. Psychiatry and clinical neurosciences 49 (5-6):291-294

2. Sivam SP, Pugazhenthi S, Pugazhenthi V, Brown H (2008) L-DOPA-induced activation of striatal p38MAPK and CREB in neonatal dopaminergic denervated rat: relevance to self-injurious behavior. Journal of neuroscience research 86 (2):339-349. doi:10.1002/jnr.21504

3. The UniProt Consortium (2017) UniProt: the universal protein knowledgebase. Nucleic Acids Research 45 (D1):D158-D169. doi:10.1093/nar/gkw1099

4. Powell JD, Lerner CG, Ewoldt GR, Schwartz RH (1999) The -180 site of the IL-2 promoter is the target of CREB/CREM binding in T cell anergy. Journal of immunology (Baltimore, Md : 1950) 163 (12):6631-6639

5. Walker WH, Daniel PB, Habener JF (1998) Inducible cAMP early repressor ICER down-regulation of CREB gene expression in Sertoli cells. Molecular and cellular endocrinology 143 (1-2):167-178

6. Masquilier D, Sassone-Corsi P (1992) Transcriptional cross-talk: nuclear factors CREM and CREB bind to AP-1 sites and inhibit activation by Jun. The Journal of biological chemistry 267 (31):22460-22466

7. Zerial M, Toschi L, Ryseck RP, Schuermann M, Muller R, Bravo R (1989) The product of a novel growth factor activated gene, fos B, interacts with JUN proteins enhancing their DNA binding activity. The EMBO journal 8 (3):805-813

8. Herdegen T, Leah JD (1998) Inducible and constitutive transcription factors in the mammalian nervous system: control of gene expression by Jun, Fos and Krox, and CREB/ATF proteins. Brain research Brain research reviews 28 (3):370-490

9. Tullai JW, Chen J, Schaffer ME, Kamenetsky E, Kasif S, Cooper GM (2007) Glycogen synthase kinase-3 represses cyclic AMP response element-binding protein (CREB)-targeted immediate early genes in quiescent cells. The Journal of biological chemistry 282 (13):9482-9491. doi:10.1074/jbc.M700067200

10. Jin G, Howe PH (1999) Transforming growth factor beta regulates clusterin gene expression via modulation of transcription factor c-Fos. European journal of biochemistry 263 (2):534-542

11. Rosenberger SF, Finch JS, Gupta A, Bowden GT (1999) Extracellular signal-regulated kinase 1/2-mediated phosphorylation of JunD and FosB is required for okadaic acid-induced activator protein 1 activation. The Journal of biological chemistry 274 (2):1124-1130

12. Kaminska B, Pyrzynska B, Ciechomska I, Wisniewska M (2000) Modulation of the composition of AP-1 complex and its impact on transcriptional activity. Acta neurobiologiae experimentalis 60 (3):395-402

13. Allan AL, Albanese C, Pestell RG, LaMarre J (2001) Activating transcription factor 3 induces DNA synthesis and expression of cyclin D1 in hepatocytes. The Journal of biological chemistry 276 (29):27272-27280. doi:10.1074/jbc.M103196200

14. MacGillavry HD, Stam FJ, Sassen MM, Kegel L, Hendriks WT, Verhaagen J, Smit AB, van Kesteren RE (2009) NFIL3 and cAMP response element-binding protein form a transcriptional feedforward loop that controls neuronal regeneration-associated gene expression. The Journal of neuroscience : the official journal of the Society for Neuroscience 29 (49):15542-15550. doi:10.1523/jneurosci.3938-09.2009

15. Leppa S, Eriksson M, Saffrich R, Ansorge W, Bohmann D (2001) Complex functions of AP-1 transcription factors in differentiation and survival of PC12 cells. Molecular and cellular biology 21 (13):4369-4378. doi:10.1128/mcb.21.13.4369-4378.2001

16. Heise CE, Reyes S, Mitrofanis J (2008) Sensory (nociceptive) stimulation evokes Fos expression in the subthalamus of hemiparkinsonian rats. Neurological research 30 (3):277-284. doi:10.1179/016164107x235455

17. Reyes S, Mitrofanis J (2008) Patterns of FOS expression in the spinal cord and periaqueductal grey matter of 6OHDA-lesioned rats. The International journal of neuroscience 118 (8):1053-1079. doi:10.1080/00207450701239210

18. Robertson GS, Herrera DG, Dragunow M, Robertson HA (1989) L-dopa activates c-fos in the striatum ipsilateral to a 6-hydroxydopamine lesion of the substantia nigra. European journal of pharmacology 159 (1):99-100

19. Cole DG, Growdon JH, DiFiglia M (1993) Levodopa induction of Fos immunoreactivity in rat brain following partial and complete lesions of the substantia nigra. Experimental neurology 120 (2):223-232. doi:10.1006/exnr.1993.1057

20. Morelli M, Cozzolino A, Pinna A, Fenu S, Carta A, Di Chiara G (1993) L-dopa stimulates c-fos expression in dopamine denervated striatum by combined activation of D-1 and D-2 receptors. Brain research 623 (2):334-336

21. Cenci MA, Tranberg A, Andersson M, Hilbertson A (1999) Changes in the regional and compartmental distribution of FosB- and JunB-like immunoreactivity induced in the dopamine-denervated rat striatum by acute or chronic L-dopa treatment. Neuroscience 94 (2):515-527

22. Winter C, Weiss C, Martin-Villalba A, Zimmermann M, Schenkel J (2002) JunB and Bcl-2 overexpression results in protection against cell death of nigral neurons following axotomy. Brain research Molecular brain research 104 (2):194-202

23. Yehia G, Schlotter F, Razavi R, Alessandrini A, Molina CA (2001) Mitogen-activated protein kinase phosphorylates and targets inducible cAMP early repressor to ubiquitin-mediated destruction. The Journal of biological chemistry 276 (38):35272-35279. doi:10.1074/jbc.M105404200

24. He Z, Jiang J, Kokkinaki M, Golestaneh N, Hofmann MC, Dym M (2008) Gdnf upregulates c-Fos transcription via the Ras/Erk1/2 pathway to promote mouse spermatogonial stem cell proliferation. Stem cells (Dayton, Ohio) 26 (1):266-278. doi:10.1634/stemcells.2007-0436

25. Byun HJ, Hong IK, Kim E, Jin YJ, Jeoung DI, Hahn JH, Kim YM, Park SH, Lee H (2006) A splice variant of CD99 increases motility and MMP-9 expression of human breast cancer cells through the AKT-, ERK-, and JNK-dependent AP-1 activation signaling pathways. The Journal of biological chemistry 281 (46):34833-34847. doi:10.1074/jbc.M605483200

26. Lutay N, Hakansson G, Alaridah N, Hallgren O, Westergren-Thorsson G, Godaly G (2014) Mycobacteria bypass mucosal NF-kB signalling to induce an epithelial anti-inflammatory IL-22 and IL-10 response. PloS one 9 (1):e86466. doi:10.1371/journal.pone.0086466

27. Nowakowski L, Kulik-Rechberger B, Wrobel A, Rechberger T (2012) [Overactive bladder--a new insight into the pathogenesis of its idiopathic form]. Ginekologia polska 83 (11):844-848

28. Bottone FG, Jr., Moon Y, Alston-Mills B, Eling TE (2005) Transcriptional regulation of activating transcription factor 3 involves the early growth response-1 gene. The Journal of pharmacology and experimental therapeutics 315 (2):668-677. doi:10.1124/jpet.105.089607

29. Colucci-D'Amato L, Perrone-Capano C, di Porzio U (2003) Chronic activation of ERK and neurodegenerative diseases. BioEssays : news and reviews in molecular, cellular and developmental biology 25 (11):1085-1095. doi:10.1002/bies.10355

30. Cheung EC, Slack RS (2004) Emerging role for ERK as a key regulator of neuronal apoptosis. Science's STKE : signal transduction knowledge environment 2004 (251):Pe45. doi:10.1126/stke.2512004pe45

31. Subramaniam S, Unsicker K (2006) Extracellular signal-regulated kinase as an inducer of non-apoptotic neuronal death. Neuroscience 138 (4):1055-1065. doi:10.1016/j.neuroscience.2005.12.013

32. Zhuang S, Schnellmann RG (2006) A death-promoting role for extracellular signal-regulated kinase. The Journal of pharmacology and experimental therapeutics 319 (3):991-997. doi:10.1124/jpet.106.107367

33. Mebratu Y, Tesfaigzi Y (2009) How ERK1/2 activation controls cell proliferation and cell death: Is subcellular localization the answer? Cell cycle (Georgetown, Tex) 8 (8):1168-1175. doi:10.4161/cc.8.8.8147

34. Subramaniam S, Unsicker K (2010) ERK and cell death: ERK1/2 in neuronal death. The FEBS journal 277 (1):22-29. doi:10.1111/j.1742-4658.2009.07367.x

35. Pavon N, Martin AB, Mendialdua A, Moratalla R (2006) ERK phosphorylation and FosB expression are associated with L-DOPA-induced dyskinesia in hemiparkinsonian mice. Biological psychiatry 59 (1):64-74. doi:10.1016/j.biopsych.2005.05.044

36. Westin JE, Vercammen L, Strome EM, Konradi C, Cenci MA (2007) Spatiotemporal pattern of striatal ERK1/2 phosphorylation in a rat model of L-DOPA-induced dyskinesia and the role of dopamine D1 receptors. Biological psychiatry 62 (7):800-810. doi:10.1016/j.biopsych.2006.11.032

37. Fiorentini C, Savoia P, Savoldi D, Barbon A, Missale C (2013) Persistent activation of the D1R/Shp-2/Erk1/2 pathway in l-DOPA-induced dyskinesia in the 6-hydroxy-dopamine rat model of Parkinson's disease. Neurobiology of disease 54:339-348. doi:10.1016/j.nbd.2013.01.005

38. Song L, Yang X, Ma Y, Wu N, Liu Z (2014) The CB1 cannabinoid receptor agonist reduces L-DOPA-induced motor fluctuation and ERK1/2 phosphorylation in 6-OHDA-lesioned rats. Drug design, development and therapy 8:2173-2179. doi:10.2147/dddt.s60944

39. Park KH, Shin KS, Zhao TT, Park HJ, Lee KE, Lee MK (2016) L-DOPA modulates cell viability through the ERK-c-Jun system in PC12 and dopaminergic neuronal cells. Neuropharmacology 101:87-97. doi:10.1016/j.neuropharm.2015.09.006

40. Zigler M, Villares GJ, Dobroff AS, Wang H, Huang L, Braeuer RR, Kamiya T, Melnikova VO, Song R, Friedman R, Alani RM, Bar-Eli M (2011) Expression of Id-1 is regulated by MCAM/MUC18: a missing link in melanoma progression. Cancer research 71 (10):3494-3504. doi:10.1158/0008-5472.can-10-3555

41. Yang J, Davies RJ, Southwood M, Long L, Yang X, Sobolewski A, Upton PD, Trembath RC, Morrell NW (2008) Mutations in bone morphogenetic protein type II receptor cause dysregulation of Id gene expression in pulmonary artery smooth muscle cells: implications for familial pulmonary arterial hypertension. Circulation research 102 (10):1212-1221. doi:10.1161/circresaha.108.173567

42. Zhang X, Ling MT, Wong YC, Wang X (2007) Evidence of a novel antiapoptotic factor: role of inhibitor of differentiation or DNA binding (Id-1) in anticancer drug-induced apoptosis. Cancer science 98 (3):308-314. doi:10.1111/j.1349-7006.2007.00400.x

43. Manrique I, Nguewa P, Bleau AM, Nistal-Villan E, Lopez I, Villalba M, Gil-Bazo I, Calvo A (2015) The inhibitor of differentiation isoform Id1b, generated by alternative splicing, maintains cell quiescence and confers self-renewal and cancer stem cell-like properties. Cancer letters 356 (2 Pt B):899-909. doi:10.1016/j.canlet.2014.10.035

44. Campbell G, Hutchins K, Winterbottom J, Grenningloh G, Lieberman AR, Anderson PN (2005) Upregulation of activating transcription factor 3 (ATF3) by intrinsic CNS neurons regenerating axons into peripheral nerve grafts. Experimental neurology 192 (2):340-347. doi:10.1016/j.expneurol.2004.11.026

45. Sodersten E, Feyder M, Lerdrup M, Gomes AL, Kryh H, Spigolon G, Caboche J, Fisone G, Hansen K (2014) Dopamine signaling leads to loss of Polycomb repression and aberrant gene activation in experimental parkinsonism. PLoS genetics 10 (9):e1004574. doi:10.1371/journal.pgen.1004574

46. Volakakis N, Kadkhodaei B, Joodmardi E, Wallis K, Panman L, Silvaggi J, Spiegelman BM, Perlmann T (2010) NR4A orphan nuclear receptors as mediators of CREB-dependent neuroprotection. Proceedings of the National Academy of Sciences of the United States of America 107 (27):12317-12322. doi:10.1073/pnas.1007088107

47. Liu TY, Yang XY, Zheng LT, Wang GH, Zhen XC (2017) Activation of Nur77 in microglia attenuates proinflammatory mediators production and protects dopaminergic neurons from inflammation-induced cell death. Journal of neurochemistry 140 (4):589-604. doi:10.1111/jnc.13907

48. Gilbert F, Morissette M, St-Hilaire M, Paquet B, Rouillard C, Di Paolo T, Levesque D (2006) Nur77 gene knockout alters dopamine neuron biochemical activity and dopamine turnover. Biological psychiatry 60 (6):538-547. doi:10.1016/j.biopsych.2006.04.023

49. St-Hilaire M, Bourhis E, Levesque D, Rouillard C (2006) Impaired behavioural and molecular adaptations to dopamine denervation and repeated L-DOPA treatment in Nur77-knockout mice. The European journal of neuroscience 24 (3):795-805. doi:10.1111/j.1460-9568.2006.04954.x

50. Mahmoudi S, Samadi P, Gilbert F, Ouattara B, Morissette M, Gregoire L, Rouillard C, Di Paolo T, Levesque D (2009) Nur77 mRNA levels and L-Dopa-induced dyskinesias in MPTP monkeys treated with docosahexaenoic acid. Neurobiology of disease 36 (1):213-222. doi:10.1016/j.nbd.2009.07.017

51. Montarolo F, Perga S, Martire S, Navone DN, Marchet A, Leotta D, Bertolotto A (2016) Altered NR4A Subfamily Gene Expression Level in Peripheral Blood of Parkinson's and Alzheimer's Disease Patients. Neurotoxicity research 30 (3):338-344. doi:10.1007/s12640-016-9626-4

52. Pearen MA, Muscat GE (2010) Minireview: Nuclear hormone receptor 4A signaling: implications for metabolic disease. Molecular endocrinology (Baltimore, Md) 24 (10):1891-1903. doi:10.1210/me.2010-0015

53. Mount MP, Zhang Y, Amini M, Callaghan S, Kulczycki J, Mao Z, Slack RS, Anisman H, Park DS (2013) Perturbation of transcription factor Nur77 expression mediated by myocyte enhancer factor 2D (MEF2D) regulates dopaminergic neuron loss in response to 1-methyl-4-phenyl-1,2,3,6-tetrahydropyridine (MPTP). The Journal of biological chemistry 288 (20):14362-14371. doi:10.1074/jbc.M112.439216

54. Lemberger T, Parkitna JR, Chai M, Schutz G, Engblom D (2008) CREB has a context-dependent role in activity-regulated transcription and maintains neuronal cholesterol homeostasis. FASEB journal : official publication of the Federation of American Societies for Experimental Biology 22 (8):2872-2879. doi:10.1096/fj.08-107888

55. Proschel C, Hansen JN, Ali A, Tuttle E, Lacagnina M, Buscaglia G, Halterman MW, Paciorkowski AR (2017) Epilepsy-causing sequence variations in SIK1 disrupt synaptic activity response gene expression and affect neuronal morphology. European journal of human genetics : EJHG 25 (2):216-221. doi:10.1038/ejhg.2016.145

56. Severini C, Improta G, Falconieri-Erspamer G, Salvadori S, Erspamer V (2002) The tachykinin peptide family. Pharmacological reviews 54 (2):285-322

57. Bourdenx M, Nilsson A, Wadensten H, Falth M, Li Q, Crossman AR, Andren PE, Bezard E (2014) Abnormal structure-specific peptide transmission and processing in a primate model of Parkinson's disease and l-DOPA-induced dyskinesia. Neurobiology of disease 62:307-312. doi:10.1016/j.nbd.2013.10.016

58. Castro-Obregon S, Rao RV, del Rio G, Chen SF, Poksay KS, Rabizadeh S, Vesce S, Zhang XK, Swanson RA, Bredesen DE (2004) Alternative, nonapoptotic programmed cell death: mediation by arrestin 2, ERK2, and Nur77. The Journal of biological chemistry 279 (17):17543-17553. doi:10.1074/jbc.M312363200

59. Saban MR, Nguyen NB, Hammond TG, Saban R (2002) Gene expression profiling of mouse bladder inflammatory responses to LPS, substance P, and antigen-stimulation. The American journal of pathology 160 (6):2095-2110. doi:10.1016/s0002-9440(10)61159-5

60. Rosati A, Graziano V, De Laurenzi V, Pascale M, Turco MC (2011) BAG3: a multifaceted protein that regulates major cell pathways. Cell death & disease 2:e141. doi:10.1038/cddis.2011.24

61. Santoro A, Nicolin V, Florenzano F, Rosati A, Capunzo M, Nori SL (2017) BAG3 is involved in neuronal differentiation and migration. Cell and tissue research 368 (2):249-258. doi:10.1007/s00441-017-2570-7

62. Lei Z, Brizzee C, Johnson GV (2015) BAG3 facilitates the clearance of endogenous tau in primary neurons. Neurobiology of aging 36 (1):241-248. doi:10.1016/j.neurobiolaging.2014.08.012

63. Bruno AP, Cefaliello C, D'Auria R, Crispino M, Rosati A, Giuditta A, Nori SL (2014) BAG3 mRNA is present in synaptosomal polysomes of rat brain. Cell cycle (Georgetown, Tex) 13 (8):1357. doi:10.4161/cc.28655

64. Gamerdinger M, Hajieva P, Kaya AM, Wolfrum U, Hartl FU, Behl C (2009) Protein quality control during aging involves recruitment of the macroautophagy pathway by BAG3. The EMBO journal 28 (7):889-901. doi:10.1038/emboj.2009.29

65. Heinrichs SC, Koob GF (2004) Corticotropin-releasing factor in brain: a role in activation, arousal, and affect regulation. The Journal of pharmacology and experimental therapeutics 311 (2):427-440. doi:10.1124/jpet.103.052092

66. Koutmani Y, Politis PK, Elkouris M, Agrogiannis G, Kemerli M, Patsouris E, Remboutsika E, Karalis KP (2013) Corticotropin-releasing hormone exerts direct effects on neuronal progenitor cells: implications for neuroprotection. Molecular psychiatry 18 (3):300-307. doi:10.1038/mp.2012.198

67. Pan JT, Lookingland KJ, Moore KE (1995) Differential Effects of Corticotropin-Releasing Hormone on Central Dopaminergic and Noradrenergic Neurons. Journal of biomedical science 2 (1):50-56

68. Bousquet C, Zatelli MC, Melmed S (2000) Direct regulation of pituitary proopiomelanocortin by STAT3 provides a novel mechanism for immuno-neuroendocrine interfacing. The Journal of clinical investigation 106 (11):1417-1425. doi:10.1172/jci11182

69. Kageyama K, Itoi K, Iwasaki Y, Niioka K, Watanuki Y, Yamagata S, Nakada Y, Das G, Suda T, Daimon M (2014) Stimulation of corticotropin-releasing factor gene expression by FosB in rat hypothalamic 4B cells. Peptides 51:59-64. doi:10.1016/j.peptides.2013.11.004

70. Parkes D, Rivest S, Lee S, Rivier C, Vale W (1993) Corticotropin-releasing factor activates c-fos, NGFI-B, and corticotropin-releasing factor gene expression within the paraventricular nucleus of the rat hypothalamus. Molecular endocrinology (Baltimore, Md) 7 (10):1357-1367. doi:10.1210/mend.7.10.8264665

71. Asadi S, Alysandratos KD, Angelidou A, Miniati A, Sismanopoulos N, Vasiadi M, Zhang B, Kalogeromitros D, Theoharides TC (2012) Substance P (SP) induces expression of functional corticotropin-releasing hormone receptor-1 (CRHR-1) in human mast cells. The Journal of investigative dermatology 132 (2):324-329. doi:10.1038/jid.2011.334

72. Rhee SH, Ma EL, Lee Y, Tache Y, Pothoulakis C, Im E (2015) Corticotropin Releasing Hormone and Urocortin 3 Stimulate Vascular Endothelial Growth Factor Expression through the cAMP/CREB Pathway. The Journal of biological chemistry 290 (43):26194-26203. doi:10.1074/jbc.M115.678979

73. Bonfiglio JJ, Inda C, Senin S, Maccarrone G, Refojo D, Giacomini D, Turck CW, Holsboer F, Arzt E, Silberstein S (2013) B-Raf and CRHR1 internalization mediate biphasic ERK1/2 activation by CRH in hippocampal HT22 Cells. Molecular endocrinology (Baltimore, Md) 27 (3):491-510. doi:10.1210/me.2012-1359

74. Rius J, Martinez-Gonzalez J, Crespo J, Badimon L (2004) Involvement of neuron-derived orphan receptor-1 (NOR-1) in LDL-induced mitogenic stimulus in vascular smooth muscle cells: role of CREB. Arteriosclerosis, thrombosis, and vascular biology 24 (4):697-702. doi:10.1161/01.ATV.0000121570.00515.dc

75. Rodriguez-Blanco J, Martin V, Herrera F, Garcia-Santos G, Antolin I, Rodriguez C (2008) Intracellular signaling pathways involved in post-mitotic dopaminergic PC12 cell death induced by 6-hydroxydopamine. Journal of neurochemistry 107 (1):127-140. doi:10.1111/j.1471-4159.2008.05588.x

76. Perlman H, Bradley K, Liu H, Cole S, Shamiyeh E, Smith RC, Walsh K, Fiore S, Koch AE, Firestein GS, Haines GK, 3rd, Pope RM (2003) IL-6 and matrix metalloproteinase-1 are regulated by the cyclin-dependent kinase inhibitor p21 in synovial fibroblasts. Journal of immunology (Baltimore, Md : 1950) 170 (2):838-845

77. Lee SJ, Cho SC, Lee EJ, Kim S, Lee SB, Lim JH, Choi YH, Kim WJ, Moon SK (2013) Interleukin-20 promotes migration of bladder cancer cells through extracellular signal-regulated kinase (ERK)-mediated MMP-9 protein expression leading to nuclear factor (NF-kappaB) activation by inducing the up-regulation of p21(WAF1) protein expression. The Journal of biological chemistry 288 (8):5539-5552. doi:10.1074/jbc.M112.410233

78. Bottazzi ME, Zhu X, Bohmer RM, Assoian RK (1999) Regulation of p21(cip1) expression by growth factors and the extracellular matrix reveals a role for transient ERK activity in G1 phase. The Journal of cell biology 146 (6):1255-1264

79. Riverso M, Montagnani V, Stecca B (2017) KLF4 is regulated by RAS/RAF/MEK/ERK signaling through E2F1 and promotes melanoma cell growth. Oncogene 36 (23):3322-3333. doi:10.1038/onc.2016.481

80. Johnson MR, Valentine C, Basilico C, Mansukhani A (1998) FGF signaling activates STAT1 and p21 and inhibits the estrogen response and proliferation of MCF-7 cells. Oncogene 16 (20):2647-2656. doi:10.1038/sj.onc.1201789

81. Sugimoto M, Martin N, Wilks DP, Tamai K, Huot TJ, Pantoja C, Okumura K, Serrano M, Hara E (2002) Activation of cyclin D1-kinase in murine fibroblasts lacking both p21(Cip1) and p27(Kip1). Oncogene 21 (53):8067-8074. doi:10.1038/sj.onc.1206019

82. Visser-Grieve S, Hao Y, Yang X (2012) Human homolog of Drosophila expanded, hEx, functions as a putative tumor suppressor in human cancer cell lines independently of the Hippo pathway. Oncogene 31 (9):1189-1195. doi:10.1038/onc.2011.318

83. Nickoloff BJ, Chaturvedi V, Bacon P, Qin JZ, Denning MF, Diaz MO (2000) Id-1 delays senescence but does not immortalize keratinocytes. The Journal of biological chemistry 275 (36):27501-27504. doi:10.1074/jbc.C000311200

84. Falco A, Festa M, Basile A, Rosati A, Pascale M, Florenzano F, Nori SL, Nicolin V, Di Benedetto M, Vecchione ML, Arra C, Barbieri A, De Laurenzi V, Turco MC (2012) BAG3 controls angiogenesis through regulation of ERK phosphorylation. Oncogene 31 (50):5153-5161. doi:10.1038/onc.2012.17

85. He G, Siddik ZH, Huang Z, Wang R, Koomen J, Kobayashi R, Khokhar AR, Kuang J (2005) Induction of p21 by p53 following DNA damage inhibits both Cdk4 and Cdk2 activities. Oncogene 24 (18):2929-2943. doi:10.1038/sj.onc.1208474

86. Copani A, Uberti D, Sortino MA, Bruno V, Nicoletti F, Memo M (2001) Activation of cell-cycle-associated proteins in neuronal death: a mandatory or dispensable path? Trends in neurosciences 24 (1):25-31

87. Sultan FA, Sweatt JD (2013) The role of the Gadd45 family in the nervous system: a focus on neurodevelopment, neuronal injury, and cognitive neuroepigenetics. Advances in experimental medicine and biology 793:81-119. doi:10.1007/978-1-4614-8289-5_6

88. Azam N, Vairapandi M, Zhang W, Hoffman B, Liebermann DA (2001) Interaction of CR6 (GADD45gamma ) with proliferating cell nuclear antigen impedes negative growth control. The Journal of biological chemistry 276 (4):2766-2774. doi:10.1074/jbc.M005626200

89. Vinayagam A, Stelzl U, Foulle R, Plassmann S, Zenkner M, Timm J, Assmus HE, Andrade-Navarro MA, Wanker EE (2011) A directed protein interaction network for investigating intracellular signal transduction. Science signaling 4 (189):rs8. doi:10.1126/scisignal.2001699

90. Obara Y, Imai T, Sato H, Takeda Y, Kato T, Ishii K (2017) Midnolin is a novel regulator of parkin expression and is associated with Parkinson's Disease. Scientific reports 7 (1):5885. doi:10.1038/s41598-017-05456-0

91. Obara Y, Ishii K (2018) Transcriptome Analysis Reveals That Midnolin Regulates mRNA Expression Levels of Multiple Parkinson's Disease Causative Genes. Biological & pharmaceutical bulletin 41 (1):20-23. doi:10.1248/bpb.b17-00663

92. Fan W, Richter G, Cereseto A, Beadling C, Smith KA (1999) Cytokine response gene 6 induces p21 and regulates both cell growth and arrest. Oncogene 18 (47):6573-6582. doi:10.1038/sj.onc.1203054

93. Kovalsky O, Lung FD, Roller PP, Fornace AJ, Jr. (2001) Oligomerization of human Gadd45a protein. The Journal of biological chemistry 276 (42):39330-39339. doi:10.1074/jbc.M105115200

94. Park HY, Ryu YK, Kim YH, Park TS, Go J, Hwang JH, Choi DH, Rhee M, Lee CH, Kim KS (2016) Gadd45beta ameliorates L-DOPA-induced dyskinesia in a Parkinson's disease mouse model. Neurobiology of disease 89:169-179. doi:10.1016/j.nbd.2016.02.013

95. Figge DA, Eskow Jaunarajs KL, Standaert DG (2016) Dynamic DNA Methylation Regulates Levodopa-Induced Dyskinesia. The Journal of neuroscience : the official journal of the Society for Neuroscience 36 (24):6514-6524. doi:10.1523/jneurosci.0683-16.2016

96. Kanaan NM, Collier TJ, Cole-Strauss A, Grabinski T, Mattingly ZR, Winn ME, Steece-Collier K, Sortwell CE, Manfredsson FP, Lipton JW (2015) The longitudinal transcriptomic response of the substantia nigra to intrastriatal 6-hydroxydopamine reveals significant upregulation of regeneration-associated genes. PloS one 10 (5):e0127768. doi:10.1371/journal.pone.0127768

97. Tamai S, Imaizumi K, Kurabayashi N, Nguyen MD, Abe T, Inoue M, Fukada Y, Sanada K (2014) Neuroprotective role of the basic leucine zipper transcription factor NFIL3 in models of amyotrophic lateral sclerosis. The Journal of biological chemistry 289 (3):1629-1638. doi:10.1074/jbc.M113.524389

98. Keniry M, Pires MM, Mense S, Lefebvre C, Gan B, Justiano K, Lau YK, Hopkins B, Hodakoski C, Koujak S, Toole J, Fenton F, Calahan A, Califano A, DePinho RA, Maurer M, Parsons R (2013) Survival factor NFIL3 restricts FOXO-induced gene expression in cancer. Genes & development 27 (8):916-927. doi:10.1101/gad.214049.113

99. Wang L, Xu S, Xu X, Chan P (2009) (-)-Epigallocatechin-3-Gallate protects SH-SY5Y cells against 6-OHDA-induced cell death through STAT3 activation. Journal of Alzheimer's disease : JAD 17 (2):295-304. doi:10.3233/jad-2009-1048

100. Kim JH, Qu A, Reddy JK, Gao B, Gonzalez FJ (2014) Hepatic oxidative stress activates the Gadd45b gene by way of degradation of the transcriptional repressor STAT3. Hepatology (Baltimore, Md) 59 (2):695-704. doi:10.1002/hep.26683

101. Scuto A, Kirschbaum M, Buettner R, Kujawski M, Cermak JM, Atadja P, Jove R (2013) SIRT1 activation enhances HDAC inhibition-mediated upregulation of GADD45G by repressing the binding of NF-kappaB/STAT3 complex to its promoter in malignant lymphoid cells. Cell death & disease 4:e635. doi:10.1038/cddis.2013.159

102. Yang F, Zhang W, Li D, Zhan Q (2013) Gadd45a suppresses tumor angiogenesis via inhibition of the mTOR/STAT3 protein pathway. The Journal of biological chemistry 288 (9):6552-6560. doi:10.1074/jbc.M112.418335

103. Lim TH, Hu L, Yang C, He C, Lee HK (2013) Membrane assisted micro-solid phase extraction of pharmaceuticals with amino and urea-grafted silica gel. Journal of chromatography A 1316:8-14. doi:10.1016/j.chroma.2013.09.034

104. Hirano T, Ishihara K, Hibi M (2000) Roles of STAT3 in mediating the cell growth, differentiation and survival signals relayed through the IL-6 family of cytokine receptors. Oncogene 19 (21):2548-2556. doi:10.1038/sj.onc.1203551

105. Coqueret O, Gascan H (2000) Functional interaction of STAT3 transcription factor with the cell cycle inhibitor p21WAF1/CIP1/SDI1. The Journal of biological chemistry 275 (25):18794-18800. doi:10.1074/jbc.M001601200

106. Li J, Bennett K, Stukalov A, Fang B, Zhang G, Yoshida T, Okamoto I, Kim JY, Song L, Bai Y, Qian X, Rawal B, Schell M, Grebien F, Winter G, Rix U, Eschrich S, Colinge J, Koomen J, Superti-Furga G, Haura EB (2013) Perturbation of the mutated EGFR interactome identifies vulnerabilities and resistance mechanisms. Molecular systems biology 9:705. doi:10.1038/msb.2013.61

107. Kong DH, Zhang Q, Meng X, Zong ZH, Li C, Liu BQ, Guan Y, Wang HQ (2013) BAG3 sensitizes cancer cells exposed to DNA damaging agents via direct interaction with GRP78. Biochimica et biophysica acta 1833 (12):3245-3253. doi:10.1016/j.bbamcr.2013.09.013

108. Ota A, Wang Y (2012) Cdc37/Hsp90 protein-mediated regulation of IRE1alpha protein activity in endoplasmic reticulum stress response and insulin synthesis in INS-1 cells. The Journal of biological chemistry 287 (9):6266-6274. doi:10.1074/jbc.M111.331264

109. Malhotra JD, Kaufman RJ (2007) The endoplasmic reticulum and the unfolded protein response. Seminars in cell & developmental biology 18 (6):716-731. doi:10.1016/j.semcdb.2007.09.003

110. Bertolotti A, Zhang Y, Hendershot LM, Harding HP, Ron D (2000) Dynamic interaction of BiP and ER stress transducers in the unfolded-protein response. Nature cell biology 2 (6):326-332. doi:10.1038/35014014

111. Salganik M, Sergeyev VG, Shinde V, Meyers CA, Gorbatyuk MS, Lin JH, Zolotukhin S, Gorbatyuk OS (2015) The loss of glucose-regulated protein 78 (GRP78) during normal aging or from siRNA knockdown augments human alpha-synuclein (alpha-syn) toxicity to rat nigral neurons. Neurobiology of aging 36 (6):2213-2223. doi:10.1016/j.neurobiolaging.2015.02.018

112. Vollmer S, Haan C, Behrmann I (2010) Oncostatin M up-regulates the ER chaperone Grp78/BiP in liver cells. Biochemical pharmacology 80 (12):2066-2073. doi:10.1016/j.bcp.2010.07.015

113. Oliveira FO, Jr., Alves CR, Souza-Silva F, Calvet CM, Cortes LM, Gonzalez MS, Toma L, Boucas RI, Nader HB, Pereira MC (2012) Trypanosoma cruzi heparin-binding proteins mediate the adherence of epimastigotes to the midgut epithelial cells of Rhodnius prolixus. Parasitology 139 (6):735-743. doi:10.1017/s0031182011002344

114. Shyu WC, Lin SZ, Chiang MF, Chen DC, Su CY, Wang HJ, Liu RS, Tsai CH, Li H (2008) Secretoneurin promotes neuroprotection and neuronal plasticity via the Jak2/Stat3 pathway in murine models of stroke. The Journal of clinical investigation 118 (1):133-148. doi:10.1172/jci32723

115. Turkson J, Bowman T, Adnane J, Zhang Y, Djeu JY, Sekharam M, Frank DA, Holzman LB, Wu J, Sebti S, Jove R (1999) Requirement for Ras/Rac1-mediated p38 and c-Jun N-terminal kinase signaling in Stat3 transcriptional activity induced by the Src oncoprotein. Molecular and cellular biology 19 (11):7519-7528

116. Xu Q, Briggs J, Park S, Niu G, Kortylewski M, Zhang S, Gritsko T, Turkson J, Kay H, Semenza GL, Cheng JQ, Jove R, Yu H (2005) Targeting Stat3 blocks both HIF-1 and VEGF expression induced by multiple oncogenic growth signaling pathways. Oncogene 24 (36):5552-5560. doi:10.1038/sj.onc.1208719

117. Li L, Hung AC, Porter AG (2008) Secretogranin II: a key AP-1-regulated protein that mediates neuronal differentiation and protection from nitric oxide-induced apoptosis of neuroblastoma cells. Cell death and differentiation 15 (5):879-888. doi:10.1038/cdd.2008.8

118. Iwase K, Ishihara A, Yoshimura S, Andoh Y, Kato M, Seki N, Matsumoto E, Hiwasa T, Muller D, Fukunaga K, Takiguchi M (2014) The secretogranin II gene is a signal integrator of glutamate and dopamine inputs. Journal of neurochemistry 128 (2):233-245. doi:10.1111/jnc.12467

119. Glauser DA, Brun T, Gauthier BR, Schlegel W (2007) Transcriptional response of pancreatic beta cells to metabolic stimulation: large scale identification of immediate-early and secondary response genes. BMC molecular biology 8:54. doi:10.1186/1471-2199-8-54

120. Soriano FX, Leveille F, Papadia S, Higgins LG, Varley J, Baxter P, Hayes JD, Hardingham GE (2008) Induction of sulfiredoxin expression and reduction of peroxiredoxin hyperoxidation by the neuroprotective Nrf2 activator 3H-1,2-dithiole-3-thione. Journal of neurochemistry 107 (2):533-543. doi:10.1111/j.1471-4159.2008.05648.x

121. Sunico CR, Sultan A, Nakamura T, Dolatabadi N, Parker J, Shan B, Han X, Yates JR, 3rd, Masliah E, Ambasudhan R, Nakanishi N, Lipton SA (2016) Role of sulfiredoxin as a peroxiredoxin-2 denitrosylase in human iPSC-derived dopaminergic neurons. Proceedings of the National Academy of Sciences of the United States of America 113 (47):E7564-e7571. doi:10.1073/pnas.1608784113

122. Koon HW, Zhao D, Na X, Moyer MP, Pothoulakis C (2004) Metalloproteinases and transforming growth factor-alpha mediate substance P-induced mitogen-activated protein kinase activation and proliferation in human colonocytes. The Journal of biological chemistry 279 (44):45519-45527. doi:10.1074/jbc.M408523200

123. Fan HY, Liu Z, Johnson PF, Richards JS (2011) CCAAT/enhancer-binding proteins (C/EBP)-alpha and -beta are essential for ovulation, luteinization, and the expression of key target genes. Molecular endocrinology (Baltimore, Md) 25 (2):253-268. doi:10.1210/me.2010-0318

124. Adderley SR, Fitzgerald DJ (1999) Oxidative damage of cardiomyocytes is limited by extracellular regulated kinases 1/2-mediated induction of cyclooxygenase-2. The Journal of biological chemistry 274 (8):5038-5046

125. Yamaguchi N, Ogawa S, Okada S (2010) Cyclooxygenase and nitric oxide synthase in the presympathetic neurons in the paraventricular hypothalamic nucleus are involved in restraint stress-induced sympathetic activation in rats. Neuroscience 170 (3):773-781. doi:10.1016/j.neuroscience.2010.07.051

126. Deng WG, Saunders M, Gilroy D, He XZ, Yeh H, Zhu Y, Shtivelband MI, Ruan KH, Wu KK (2002) Purification and characterization of a cyclooxygenase-2 and angiogenesis suppressing factor produced by human fibroblasts. FASEB journal : official publication of the Federation of American Societies for Experimental Biology 16 (10):1286-1288. doi:10.1096/fj.01-0844fje

127. Ozkurt IC, Tetradis S (2003) Parathyroid hormone-induced E4BP4/NFIL3 down-regulates transcription in osteoblasts. The Journal of biological chemistry 278 (29):26803-26809. doi:10.1074/jbc.M212652200

128. Ludwig A, Uvarov P, Pellegrino C, Thomas-Crusells J, Schuchmann S, Saarma M, Airaksinen MS, Rivera C (2011) Neurturin evokes MAPK-dependent upregulation of Egr4 and KCC2 in developing neurons. Neural plasticity 2011:1-8. doi:10.1155/2011/641248

129. Charbonnier-Beaupel F, Malerbi M, Alcacer C, Tahiri K, Carpentier W, Wang C, During M, Xu D, Worley PF, Girault JA, Herve D, Corvol JC (2015) Gene expression analyses identify Narp contribution in the development of L-DOPA-induced dyskinesia. The Journal of neuroscience : the official journal of the Society for Neuroscience 35 (1):96-111. doi:10.1523/jneurosci.5231-13.2015

130. Spiegel I, Mardinly AR, Gabel HW, Bazinet JE, Couch CH, Tzeng CP, Harmin DA, Greenberg ME (2014) Npas4 regulates excitatory-inhibitory balance within neural circuits through cell-type-specific gene programs. Cell 157 (5):1216-1229. doi:10.1016/j.cell.2014.03.058

131. Moran LB, Hickey L, Michael GJ, Derkacs M, Christian LM, Kalaitzakis ME, Pearce RK, Graeber MB (2008) Neuronal pentraxin II is highly upregulated in Parkinson's disease and a novel component of Lewy bodies. Acta neuropathologica 115 (4):471-478. doi:10.1007/s00401-007-0309-3

132. Bjartmar L, Huberman AD, Ullian EM, Renteria RC, Liu X, Xu W, Prezioso J, Susman MW, Stellwagen D, Stokes CC, Cho R, Worley P, Malenka RC, Ball S, Peachey NS, Copenhagen D, Chapman B, Nakamoto M, Barres BA, Perin MS (2006) Neuronal pentraxins mediate synaptic refinement in the developing visual system. The Journal of neuroscience : the official journal of the Society for Neuroscience 26 (23):6269-6281. doi:10.1523/jneurosci.4212-05.2006

133. Rui L, Fisher TL, Thomas J, White MF (2001) Regulation of insulin/insulin-like growth factor-1 signaling by proteasome-mediated degradation of insulin receptor substrate-2. The Journal of biological chemistry 276 (43):40362-40367. doi:10.1074/jbc.M105332200

134. Russo SJ, Bolanos CA, Theobald DE, DeCarolis NA, Renthal W, Kumar A, Winstanley CA, Renthal NE, Wiley MD, Self DW, Russell DS, Neve RL, Eisch AJ, Nestler EJ (2007) IRS2-Akt pathway in midbrain dopamine neurons regulates behavioral and cellular responses to opiates. Nature neuroscience 10 (1):93-99. doi:10.1038/nn1812

135. Morris JK, Zhang H, Gupte AA, Bomhoff GL, Stanford JA, Geiger PC (2008) Measures of striatal insulin resistance in a 6-hydroxydopamine model of Parkinson's disease. Brain research 1240:185-195. doi:10.1016/j.brainres.2008.08.089

136. Karbowniczek M, Cash T, Cheung M, Robertson GP, Astrinidis A, Henske EP (2004) Regulation of B-Raf kinase activity by tuberin and Rheb is mammalian target of rapamycin (mTOR)-independent. The Journal of biological chemistry 279 (29):29930-29937. doi:10.1074/jbc.M402591200

137. Shah OJ, Wang Z, Hunter T (2004) Inappropriate activation of the TSC/Rheb/mTOR/S6K cassette induces IRS1/2 depletion, insulin resistance, and cell survival deficiencies. Current biology : CB 14 (18):1650-1656. doi:10.1016/j.cub.2004.08.026

138. Kim SR, Kareva T, Yarygina O, Kholodilov N, Burke RE (2012) AAV transduction of dopamine neurons with constitutively active Rheb protects from neurodegeneration and mediates axon regrowth. Molecular therapy : the journal of the American Society of Gene Therapy 20 (2):275-286. doi:10.1038/mt.2011.213

139. Kim SR, Chen X, Oo TF, Kareva T, Yarygina O, Wang C, During M, Kholodilov N, Burke RE (2011) Dopaminergic pathway reconstruction by Akt/Rheb-induced axon regeneration. Annals of neurology 70 (1):110-120. doi:10.1002/ana.22383

140. Jeon MT, Kim SR (2015) Roles of Rheb(S16H) in substantia nigra pars compacta dopaminergic neurons in vivo. Biomedical reports 3 (2):137-140. doi:10.3892/br.2014.397

141. Yang CY, Li JP, Chiu LL, Lan JL, Chen DY, Chuang HC, Huang CY, Tan TH (2014) Dual-specificity phosphatase 14 (DUSP14/MKP6) negatively regulates TCR signaling by inhibiting TAB1 activation. Journal of immunology (Baltimore, Md : 1950) 192 (4):1547-1557. doi:10.4049/jimmunol.1300989

142. Huang CY, Tan TH (2012) DUSPs, to MAP kinases and beyond. Cell & bioscience 2 (1):24. doi:10.1186/2045-3701-2-24

143. Heiman M, Heilbut A, Francardo V, Kulicke R, Fenster RJ, Kolaczyk ED, Mesirov JP, Surmeier DJ, Cenci MA, Greengard P (2014) Molecular adaptations of striatal spiny projection neurons during levodopa-induced dyskinesia. Proceedings of the National Academy of Sciences of the United States of America 111 (12):4578-4583. doi:10.1073/pnas.1401819111

144. Gerfen CR, Miyachi S, Paletzki R, Brown P (2002) D1 dopamine receptor supersensitivity in the dopamine-depleted striatum results from a switch in the regulation of ERK1/2/MAP kinase. The Journal of neuroscience : the official journal of the Society for Neuroscience 22 (12):5042-5054

145. Chevrier N, Mertins P, Artyomov MN, Shalek AK, Iannacone M, Ciaccio MF, Gat-Viks I, Tonti E, DeGrace MM, Clauser KR, Garber M, Eisenhaure TM, Yosef N, Robinson J, Sutton A, Andersen MS, Root DE, von Andrian U, Jones RB, Park H, Carr SA, Regev A, Amit I, Hacohen N (2011) Systematic discovery of TLR signaling components delineates viral-sensing circuits. Cell 147 (4):853-867. doi:10.1016/j.cell.2011.10.022

146. Centonze D, Napolitano M, Saulle E, Gubellini P, Picconi B, Martorana A, Pisani A, Gulino A, Bernardi G, Calabresi P (2002) Tissue plasminogen activator is required for corticostriatal long-term potentiation. The European journal of neuroscience 16 (4):713-721

147. Hebert M, Lesept F, Vivien D, Macrez R (2016) The story of an exceptional serine protease, tissue-type plasminogen activator (tPA). Revue neurologique 172 (3):186-197. doi:10.1016/j.neurol.2015.10.002

148. Shav-Tal Y, Zipori D (2002) The role of activin a in regulation of hemopoiesis. Stem cells (Dayton, Ohio) 20 (6):493-500. doi:10.1634/stemcells.20-6-493

149. Mantuano E, Lam MS, Gonias SL (2013) LRP1 assembles unique co-receptor systems to initiate cell signaling in response to tissue-type plasminogen activator and myelin-associated glycoprotein. The Journal of biological chemistry 288 (47):34009-34018. doi:10.1074/jbc.M113.509133

150. Poulaki V, Mitsiades N, Kruse FE, Radetzky S, Iliaki E, Kirchhof B, Joussen AM (2004) Activin a in the regulation of corneal neovascularization and vascular endothelial growth factor expression. The American journal of pathology 164 (4):1293-1302. doi:10.1016/s0002-9440(10)63216-6

151. Hashimoto M, Gaddy-Kurten D, Vale W (1993) Protooncogene junB as a target for activin actions. Endocrinology 133 (5):1934-1940. doi:10.1210/endo.133.5.8404639

152. Seko Y, Takahashi N, Tobe K, Ueki K, Kadowaki T, Yazaki Y (1998) Vascular endothelial growth factor (VEGF) activates Raf-1, mitogen-activated protein (MAP) kinases, and S6 kinase (p90rsk) in cultured rat cardiac myocytes. Journal of cellular physiology 175 (3):239-246. doi:10.1002/(sici)1097-4652(199806)175:3<239::aid-jcp1>3.0.co;2-p

153. Kuba K, Matsumoto K, Date K, Shimura H, Tanaka M, Nakamura T (2000) HGF/NK4, a four-kringle antagonist of hepatocyte growth factor, is an angiogenesis inhibitor that suppresses tumor growth and metastasis in mice. Cancer research 60 (23):6737-6743

154. Liu Y, Mueller BM (2006) Protease-activated receptor-2 regulates vascular endothelial growth factor expression in MDA-MB-231 cells via MAPK pathways. Biochemical and biophysical research communications 344 (4):1263-1270. doi:10.1016/j.bbrc.2006.04.005

155. Seghezzi G, Patel S, Ren CJ, Gualandris A, Pintucci G, Robbins ES, Shapiro RL, Galloway AC, Rifkin DB, Mignatti P (1998) Fibroblast growth factor-2 (FGF-2) induces vascular endothelial growth factor (VEGF) expression in the endothelial cells of forming capillaries: an autocrine mechanism contributing to angiogenesis. The Journal of cell biology 141 (7):1659-1673

156. Goldin LR, Martinez MM (1989) The detection of linkage and heterogeneity in nuclear families when unaffected individuals are considered unknown. Progress in clinical and biological research 329:195-200

157. Herran E, Requejo C, Ruiz-Ortega JA, Aristieta A, Igartua M, Bengoetxea H, Ugedo L, Pedraz JL, Lafuente JV, Hernandez RM (2014) Increased antiparkinson efficacy of the combined administration of VEGF- and GDNF-loaded nanospheres in a partial lesion model of Parkinson's disease. International journal of nanomedicine 9:2677-2687. doi:10.2147/ijn.s61940

158. Holmes DI, Zachary I (2004) Placental growth factor induces FosB and c-Fos gene expression via Flt-1 receptors. FEBS letters 557 (1-3):93-98

159. Rius J, Martinez-Gonzalez J, Crespo J, Badimon L (2006) NOR-1 is involved in VEGF-induced endothelial cell growth. Atherosclerosis 184 (2):276-282. doi:10.1016/j.atherosclerosis.2005.04.008

160. Barua RS, Ambrose JA, Saha DC, Eales-Reynolds LJ (2002) Smoking is associated with altered endothelial-derived fibrinolytic and antithrombotic factors: an in vitro demonstration. Circulation 106 (8):905-908

161. Huber D, Cramer EM, Kaufmann JE, Meda P, Masse JM, Kruithof EK, Vischer UM (2002) Tissue-type plasminogen activator (t-PA) is stored in Weibel-Palade bodies in human endothelial cells both in vitro and in vivo. Blood 99 (10):3637-3645

162. Ohnesorge N, Viemann D, Schmidt N, Czymai T, Spiering D, Schmolke M, Ludwig S, Roth J, Goebeler M, Schmidt M (2010) Erk5 activation elicits a vasoprotective endothelial phenotype via induction of Kruppel-like factor 4 (KLF4). The Journal of biological chemistry 285 (34):26199-26210. doi:10.1074/jbc.M110.103127

163. Matys T, Pawlak R, Matys E, Pavlides C, McEwen BS, Strickland S (2004) Tissue plasminogen activator promotes the effects of corticotropin-releasing factor on the amygdala and anxiety-like behavior. Proceedings of the National Academy of Sciences of the United States of America 101 (46):16345-16350. doi:10.1073/pnas.0407355101

164. Santos-Carvalho A, Elvas F, Alvaro AR, Ambrosio AF, Cavadas C (2013) Neuropeptide Y receptors activation protects rat retinal neural cells against necrotic and apoptotic cell death induced by glutamate. Cell death & disease 4:e636. doi:10.1038/cddis.2013.160

165. Ferreira-Marques M, Aveleira CA, Carmo-Silva S, Botelho M, Pereira de Almeida L, Cavadas C (2016) Caloric restriction stimulates autophagy in rat cortical neurons through neuropeptide Y and ghrelin receptors activation. Aging 8 (7):1470-1484. doi:10.18632/aging.100996

166. Hansel DE, Eipper BA, Ronnett GV (2001) Neuropeptide Y functions as a neuroproliferative factor. Nature 410 (6831):940-944. doi:10.1038/35073601

167. Decressac M, Pain S, Chabeauti PY, Frangeul L, Thiriet N, Herzog H, Vergote J, Chalon S, Jaber M, Gaillard A (2012) Neuroprotection by neuropeptide Y in cell and animal models of Parkinson's disease. Neurobiology of aging 33 (9):2125-2137. doi:10.1016/j.neurobiolaging.2011.06.018

168. Liu Y, Poon V, Sanchez-Watts G, Watts AG, Takemori H, Aguilera G (2012) Salt-inducible kinase is involved in the regulation of corticotropin-releasing hormone transcription in hypothalamic neurons in rats. Endocrinology 153 (1):223-233. doi:10.1210/en.2011-1404

169. Irie Y, Yamagata K, Gan Y, Miyamoto K, Do E, Kuo CH, Taira E, Miki N (2000) Molecular cloning and characterization of Amida, a novel protein which interacts with a neuron-specific immediate early gene product arc, contains novel nuclear localization signals, and causes cell death in cultured cells. The Journal of biological chemistry 275 (4):2647-2653

170. Myrum C, Baumann A, Bustad HJ, Flydal MI, Mariaule V, Alvira S, Cuellar J, Haavik J, Soule J, Valpuesta JM, Marquez JA, Martinez A, Bramham CR (2015) Arc is a flexible modular protein capable of reversible self-oligomerization. The Biochemical journal 468 (1):145-158. doi:10.1042/bj20141446

171. Sgambato-Faure V, Buggia V, Gilbert F, Levesque D, Benabid AL, Berger F (2005) Coordinated and spatial upregulation of arc in striatonigral neurons correlates with L-dopa-induced behavioral sensitization in dyskinetic rats. Journal of neuropathology and experimental neurology 64 (11):936-947

172. Garcia PC, Real CC, Britto LR (2017) The Impact of Short and Long-Term Exercise on the Expression of Arc and AMPARs During Evolution of the 6-Hydroxy-Dopamine Animal Model of Parkinson's Disease. Journal of molecular neuroscience : MN 61 (4):542-552. doi:10.1007/s12031-017-0896-y

173. Nikolaienko O, Eriksen MS, Patil S, Bito H, Bramham CR (2017) Stimulus-evoked ERK-dependent phosphorylation of activity-regulated cytoskeleton-associated protein (Arc) regulates its neuronal subcellular localization. Neuroscience 360:68-80. doi:10.1016/j.neuroscience.2017.07.026

174. Menard C, Tse YC, Cavanagh C, Chabot JG, Herzog H, Schwarzer C, Wong TP, Quirion R (2013) Knockdown of prodynorphin gene prevents cognitive decline, reduces anxiety, and rescues loss of group 1 metabotropic glutamate receptor function in aging. The Journal of neuroscience : the official journal of the Society for Neuroscience 33 (31):12792-12804. doi:10.1523/jneurosci.0290-13.2013

175. Wang Y, Ju W, Liu L, Fam S, D'Souza S, Taghibiglou C, Salter M, Wang YT (2004) alpha-Amino-3-hydroxy-5-methylisoxazole-4-propionic acid subtype glutamate receptor (AMPAR) endocytosis is essential for N-methyl-D-aspartate-induced neuronal apoptosis. The Journal of biological chemistry 279 (40):41267-41270. doi:10.1074/jbc.C400199200

176. Cenci MA, Lee CS, Bjorklund A (1998) L-DOPA-induced dyskinesia in the rat is associated with striatal overexpression of prodynorphin- and glutamic acid decarboxylase mRNA. The European journal of neuroscience 10 (8):2694-2706

177. Hanrieder J, Ljungdahl A, Falth M, Mammo SE, Bergquist J, Andersson M (2011) L-DOPA-induced dyskinesia is associated with regional increase of striatal dynorphin peptides as elucidated by imaging mass spectrometry. Molecular & cellular proteomics : MCP 10 (10):M111.009308. doi:10.1074/mcp.M111.009308

178. Chen Z, Guan Q, Cao X, Xu Y, Wang L, Sun S (2006) Effect of antisense FosB and CREB on the expression of prodynorphin gene in rats with levodopa-induced dyskinesias. Journal of Huazhong University of Science and Technology Medical sciences = Hua zhong ke ji da xue xue bao Yi xue Ying De wen ban = Huazhong keji daxue xuebao Yixue Yingdewen ban 26 (5):542-544

179. Morioka N, Sugimoto T, Sato K, Okazaki S, Saeki M, Hisaoka-Nakashima K, Nakata Y (2015) The induction of Per1 expression by the combined treatment with glutamate, 5-hydroxytriptamine and dopamine initiates a ripple effect on Bmal1 and Cry1 mRNA expression via the ERK signaling pathway in cultured rat spinal astrocytes. Neurochemistry international 90:9-19. doi:10.1016/j.neuint.2015.06.013

180. Balsalobre A, Marcacci L, Schibler U (2000) Multiple signaling pathways elicit circadian gene expression in cultured Rat-1 fibroblasts. Current biology : CB 10 (20):1291-1294

181. Wu QF, Yang L, Li S, Wang Q, Yuan XB, Gao X, Bao L, Zhang X (2012) Fibroblast growth factor 13 is a microtubule-stabilizing protein regulating neuronal polarization and migration. Cell 149 (7):1549-1564. doi:10.1016/j.cell.2012.04.046

182. Lu H, Shi X, Wu G, Zhu J, Song C, Zhang Q, Yang G (2015) FGF13 regulates proliferation and differentiation of skeletal muscle by down-regulating Spry1. Cell proliferation 48 (5):550-560. doi:10.1111/cpr.12200
